# Marine diatoms change their gene expression profile when exposed to microscale turbulence under nutrient replete conditions

**DOI:** 10.1038/s41598-017-03741-6

**Published:** 2017-06-19

**Authors:** Alberto Amato, Gianluca Dell’Aquila, Francesco Musacchia, Rossella Annunziata, Ari Ugarte, Nicolas Maillet, Alessandra Carbone, Maurizio Ribera d’Alcalà, Remo Sanges, Daniele Iudicone, Maria I. Ferrante

**Affiliations:** 1 Stazione Zoologica Anton Dohrn, Villa Comunale, 80121 Naples, Italy; 2 Sorbonne Universités, UPMC Univ-Paris 6, CNRS, UMR 7238, Laboratoire de Biologie Computationnelle et Quantitative, 15 rue de l’Ecole de Médecine, 75006 Paris, France; 3 Institut Universitaire de France, 75005 Paris, France; 4 Laboratoire de Physiologie Cellulaire et Végétale, UMR5168 CNRS-CEA-INRA-Université de Grenoble Alpes, Institut de Recherche en Science et Technologies pour le Vivant, CEA Grenoble, 17 rue des Martyrs, 38054 Grenoble Cédex 9, France; 5 Zellbiologie Philipps-Universität Marburg, Karl-von-Frisch Str, 8 35043 Marburg, Germany; 6 Telethon Institute for Genetics and Medicine (TIGEM), Viale Campi Flegrei 34, 80078 Pozzuoli - Naples, Italy; 7 Institut Pasteur – Bioinformatics and Biostatistics Hub – C3BI – CNRS, USR 3756 25-28 Rue du Dr Roux, 75015 Paris, France

## Abstract

Diatoms are a fundamental microalgal phylum that thrives in turbulent environments. Despite several experimental and numerical studies, if and how diatoms may profit from turbulence is still an open question. One of the leading arguments is that turbulence favours nutrient uptake. Morphological features, such as the absence of flagella, the presence of a rigid exoskeleton and the micrometre size would support the possible passive but beneficial role of turbulence on diatoms. We demonstrate that in fact diatoms actively respond to turbulence in non-limiting nutrient conditions. TURBOGEN, a prototypic instrument to generate natural levels of microscale turbulence, was used to expose diatoms to the mechanical stimulus. Differential expression analyses, coupled with microscopy inspections, enabled us to study the morphological and transcriptional response of *Chaetoceros decipiens* to turbulence. Our target species responds to turbulence by activating energy storage pathways like fatty acid biosynthesis and by modifying its cell chain spectrum. Two other ecologically important species were examined and the occurrence of a morphological response was confirmed. These results challenge the view of phytoplankton as unsophisticated passive organisms.

## Introduction

Oceanic plankton are characterised by a huge biodiversity spanning over many phyla. The different life strategies and behaviours displayed by such diverse organisms were selected also to cope with a patchy and variable 3D world driven by water motion^1^. Key factors for their survival, e.g., light availability, nutrient concentrations, prey abundance and water temperature, are all strongly tuned by the fluid motion at different scales. Fluid motion introduces kinetic energy in the system and turbulence is the way kinetic energy is transferred, through a dissipation cascade, over several eddy-like structures down to the smallest scale. Below this scale, energy is dissipated to heat via the friction of viscosity and water motion cannot prevail over molecular diffusion but can control it by changing local gradients^2, 3^. This is particularly important for unicellular phytoplankton which are surrounded by a fluid boundary layer where molecular diffusion is the dominant process and only solute (nutrient) chemical gradients assure cell provisioning. A distortion of the boundary layer would change these gradients, hence nutrients would diffuse more rapidly, enhancing the uptake rate^4^. For non-motile cells, like diatoms, the distortion of the boundary layer can be produced only by sinking or by the shear generated by the decay of turbulent kinetic energy. Therefore there is no more turbulence, as a random motion of water parcels, but the effects of turbulence which dissipates though sheared flow. Shear stress is what cells below Kolmogorov scale would ultimately perceive in a turbulent environment. Microturbulence may also favour unicellular autotrophs, bringing them into the upper illuminated layer of the ocean, the euphotic zone^5^. The arguments above, together with cell size and the ability to produce chains^6–10^, are frequently invoked to explain why diatoms should be favoured in turbulent environments^11, 12^.

Mechanistic studies^4, 13^ predict that diatoms could profit from turbulent pulses, even without any physiological adjustment. We note that following these studies some of the necessary conditions for an impact of microturbulence on diatoms, i.e., intense turbulence, nutrient depletion and grazing pressure^14^, are infrequently met in the oceans^15, 16^. Thus, on the basis of current theories, diatoms would not specifically be adapted to microscale turbulence^14^. Nonetheless, that diatoms can sense mechanical stimuli was demonstrated by shaking a suspension of a *Phaeodactylum tricornutum* aequorin transformant with a needle and observing cytosolic calcium increases after 1–2 seconds after application of the stimulus and declining soon after^17^. Cytosolic calcium waves trigger the activation of signal transduction^18^ i.e. a response to a perceived stimulus. This raises the question if turbulence produces changes on some proximal environmental variables which would initiate diatom response similarly to what happens for light^19–21^ or if turbulence acts as a signal, carrying information about the environmental context that diatoms exploit to rearrange their physiology. Considering that the effects of physical forcing can propagate from single cells through populations into marine ecosystems^22^ and that diatoms are considered among the most productive phyla in the world ocean^23, 24^, a controlled response of diatoms to fluid motion would imply that the whole food web can be affected by small scale turbulence^25^ with repercussions on the whole ecosystem^26^.

In the present study, we asked whether turbulence could trigger a response in diatoms when nutrients were in excess and all other environmental parameters were kept identical to control still samples. To this aim we applied measured levels of turbulence, in the order of those found in the field^27^, to the diatom *Chaetoceros decipiens* using a prototypic turbulence generator (TURBOGEN^28^) and performed both microscopic inspection of chain lengths and transcriptomic analyses. Chain formation is often put in relation to turbulence^13^ and *C. decipiens* displays the peculiar trait of being able to control chain length^29^. Moreover this species belongs to the most abundant diatom genus in ocean waters^30^. We conducted the experiments in natural cell concentrations and in nutrient replete condition to rule out responses due to over-crowding and starvation stresses.

Our results showed that *C. decipiens* responded both morphologically and at the gene expression level to the treatment. The transcriptomic response was mild as expected because turbulence is not a stressor like nutrient starvation^31–34^, high irradiance^35^ or chemicals^36^. Unexpectedly, this species produced shorter chains in turbulence; in fact chain length is correlated to cell growth and in our experiments, cultures were actively growing. We also extended our morphological analysis to two other centric diatoms, *Thalassiosira rotula* and *Skeletonema marinoi*, which show different chain formation modes, to assess whether the morphological response observed in *C. decipiens* followed a general pattern or was species-specific. We hypothesise that diatoms do not just profit from turbulence pulses, but likely reorganise their physiology and morphology in presence of turbulence.

## Results

### Reference *de novo* assembled transcriptome

RNA-seq data (Supplementary Fig. 1, Supplementary Information) were used to produce a reference transcriptome for *Chaetoceros decipiens*. The Illumina sequencing output and *de novo* transcriptome assembly statistics are summarised in Table 1. The transcriptome contained ∼28,000 contigs and ∼21,000 unique transcripts (Supplementary Information), i.e. twice as many as *Thalassiosira pseudonana* ^37^ and *Phaeodactylum tricornutum* ^38^ genomes, but similar to other *de novo* diatom transcriptomes^39^, including *Chaetoceros* species^40^. More than half of the transcripts were annotated using different algorithms (Supplementary Fig. 2, Supplementary Information).

**Table 1:** Transcriptome statistics and differential analyses outcomes.

**Illumina sequencing output**				
# of reads		Read length		
2.3·10^8^		50 bp		
***De novo*** **transcriptome assembly**				
Trinity contigs	Unique transcripts	Average length	Minimum length	Maximum length
27923	21224	1496 bp	201 bp	11631 bp
**Annotation**				
Annocript annotation		META-CLADE domains		HMM Scan domains
69.38%		20523		15101
**Differential expression analyses**				
T2			T3	
1330 UP-reg			230 UP-reg	
6.3%			1.1%	
1297 DOWN-reg			243 DOWN-reg	
6.1%			1.1%	

### Gene expression changes in cultures exposed to turbulence

In order to assess whether *C. decipiens* cells exposed to turbulence experienced changes in their gene expression profile, a low density culture was aliquoted in the six TURBOGEN cylinders, turbulence was applied to three cylinders and samples were collected from still and turbulent cylinders at 48 and 72 hours from the beginning of the experiment (time points T2 and T3, respectively, Supplementary Fig. 1).

**Figure 1:**
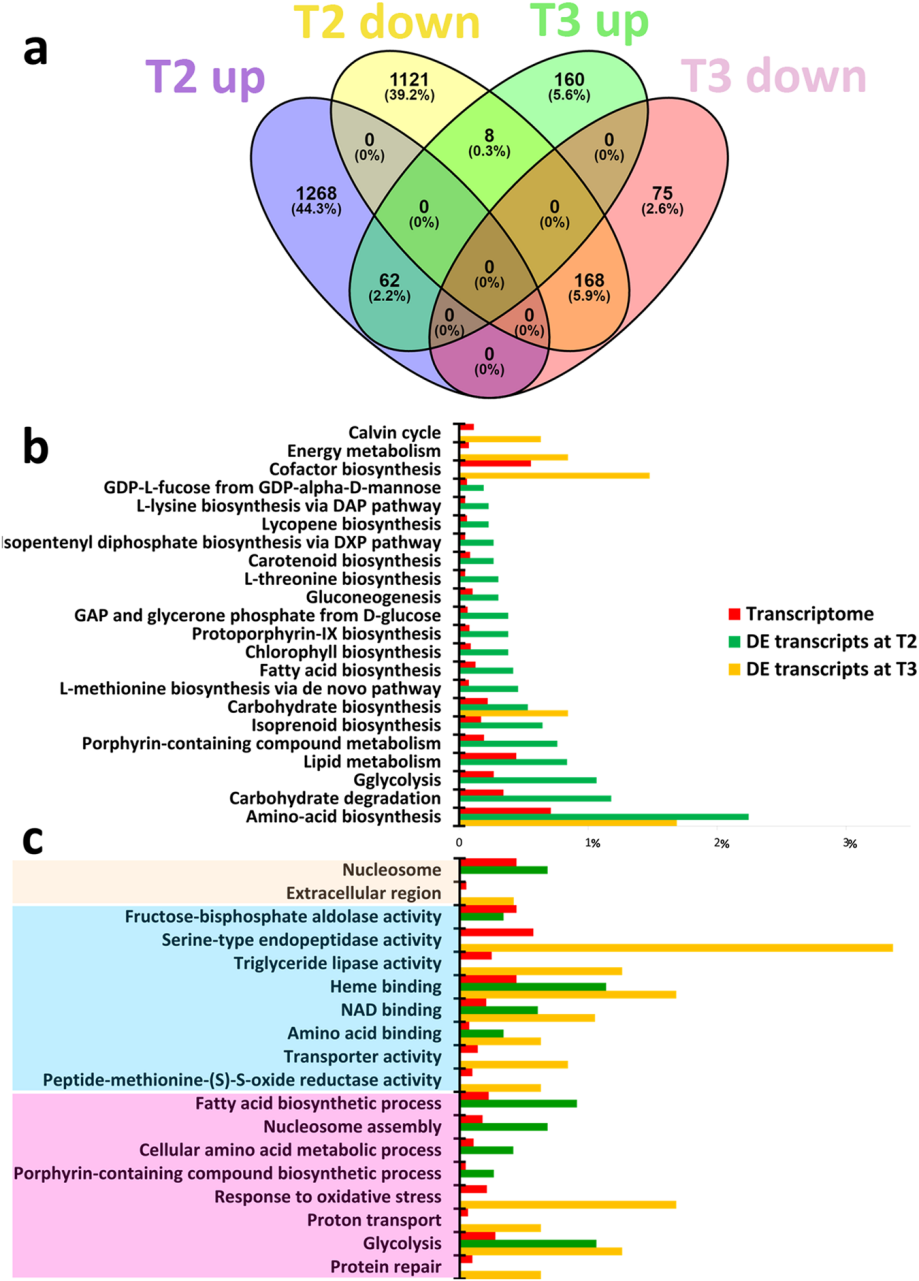
Differential expression analyses in *Chaetoceros decipiens*. (**a**) Venn diagram of DE transcripts at time points T2 and T3. Number of transcripts and percentage are shown. Venn diagrams have been produced using Venny 2.0 online software^71^. (**b**,**c**) Representation of (**b**) KO pathway level 1 and (**c**) GO terms significantly enriched for DE transcripts. The charts show the proportion of transcripts associated to the significant classes for DE transcripts at time points T2 (green) and T3 (yellow) and their respective proportion in the assembled reference transcriptome (red). Colour shades indicate cellular components (ochre); biological processes (blue); molecular function (pink). On the x-axis, the percentage of transcripts associated to a given KO (**b**) or to a specific GO term (**c**) calculated over the total number of transcripts in the given dataset is reported.

About 12% (T2) and ∼2% (T3) of the total *C. decipiens* transcripts were differentially expressed (DE, Supplementary Tables 1–3), of which the vast majority were unique to T2 (Fig. 1a). RNA-seq results were validated by qPCR using independent RNA samples (Supplementary Tables 4 and 5).

In KO pathway (level 1, Fig. 1b) and GO-term (Fig. 1c) enrichment analyses the frequency of a given term in the entire transcriptome was compared with frequency of the same term in the DE transcript dataset, showing that a number of functions were significantly enriched in the latter. This is an indication that these functions might be involved in turbulence perception and/or response. This result was corroborated by plotting onto KEGG pathways the DE datasets for T2 (Supplementary Fig. 3a) and T3 (Supplementary Fig. 3b). The most regulated pathways were Calvin cycle, fatty acid (FA) biosynthesis, glycolysis and pentose phosphate pathway (PPP) (Supplementary Table 3). Transcripts from the inositol and methylerythritol phosphate (isoprenoid biosynthesis) pathways were regulated in presence of turbulence (Supplementary Table 6). Three transcripts annotated as nucleotide diphosphate protein kinase (NDPK) were up-regulated in turbulent condition (Supplementary Table 6). FA biosynthesis requires, besides substrates, reducing power in the form of NADPH^+^ that can be provided by the pentose phosphate pathway (PPP), which was up-regulated in our experiments. The differential expression analysis indicated that PPP produced NADPH^+^ and GAP with the ribulose-5-phosphate preferentially following the non-oxidative way of the PPP. GAP is the substrate for one of the two isoprenoid biosynthesis pathways, namely the methylerythritol phosphate pathway (MEP) that in turbulence-exposed cultures presented seven out of the nine transcripts up-regulated.

### Morphological changes in diatom cultures exposed to turbulence

**Figure 2:**
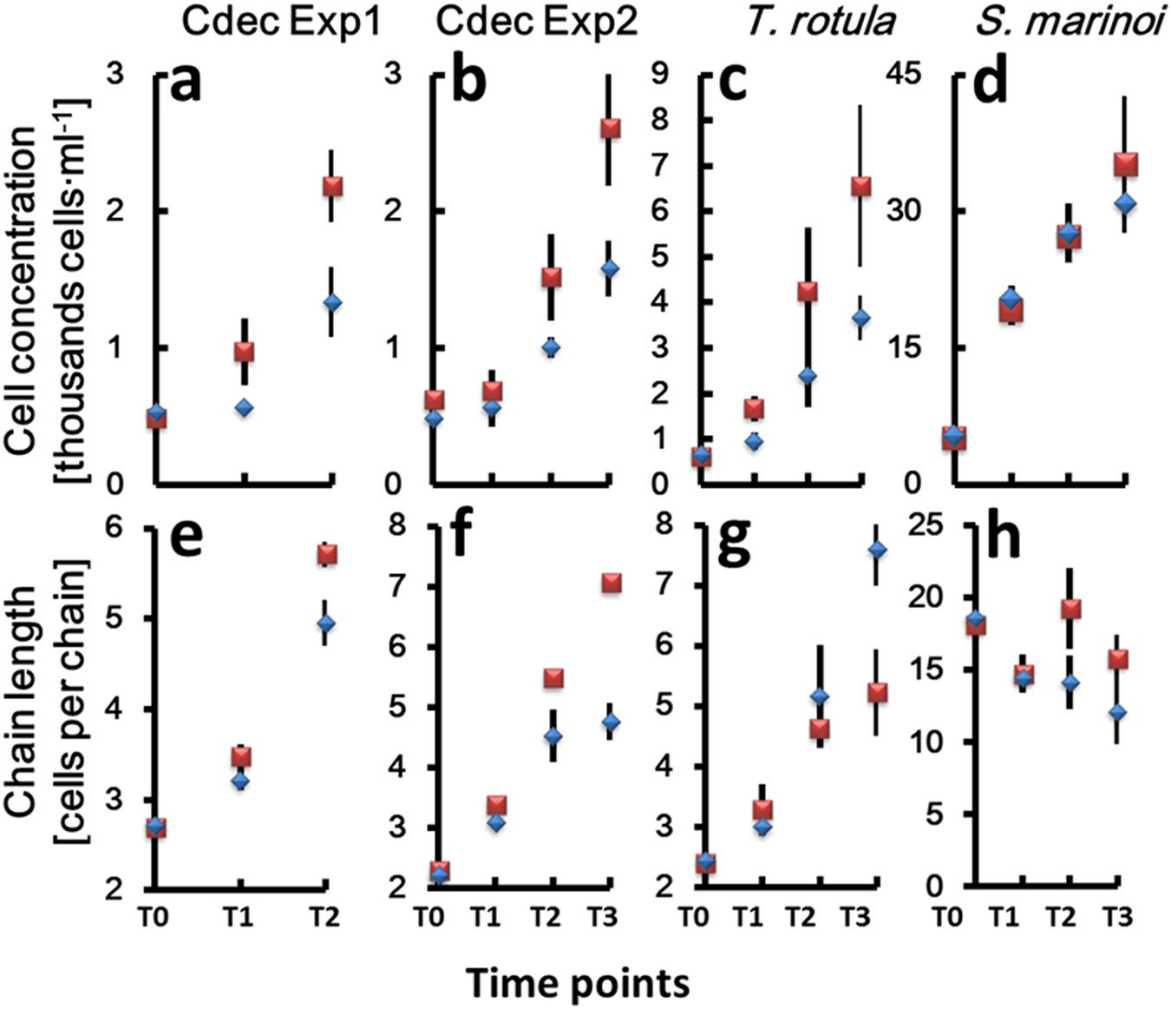
Growth curves expressed as cells · ml^−1^ (y-axis) (**a–d**) and mean chain length expressed as number of cells per chain (y-axis) (**e**–**h**) over the time points (x-axis). (**a**,**e**) *Chaetoceros decipiens* experiment 1; (**b**,**f**) *Chaetoceros decipiens* experiment 2; (**c**,**g**) *Thalassiosira rotula*; (**d**,**h**) *Skeletonema marinoi*. Blue diamonds = turbulence exposed cells. Red squares = still condition. Vertical lines indicate standard deviation. Values have been averaged over the three replicates.

Besides RNA extraction and sequencing, culture samples were collected for microscopic inspection and cell counts every 24 hours from the beginning of the experiment (Supplementary Fig. 1). In addition to the experiments with *C. decipiens*, we used the TURBOGEN with the same experimental set up to study the response of two more chain-forming diatoms, *Thalassiosira rotula* and *Skeletonema marinoi*. Cell counts (Supplementary Table 7, Fig. 2a–d) and chain length measurements were performed (Fig. 2e–h) and division rates calculated. *C. decipiens* cultures presented a one-day lag phase of growth (Fig. 2a,b) and this behaviour was recorded also in *T. rotula* exposed to turbulence (Fig. 2c). *S. marinoi* did not show such a lag phase (Fig. 2d). Overall, division rates between control and treated cultures were comparable (Table 2), with slightly different values recorded only for *C. decipiens* in experiment 2.

**Table 2:** Division rates calculated as the first derivative of the steepest portion of the growth curve. Average ± standard deviation is reported (n = 3).

Species	Division rate	
	Turbulent	Still
*C. decipiens* Exp 1	1.2 ± 0.20	1.2 ± 0.54
*C. decipiens* Exp 2	0.8 ± 0.27	1.1 ± 0.14
*T. rotula*	1.0 ± 0.08	1.0 ± 0.17
*S. marinoi*	1.2 ± 0.11	1.2 ± 0.01

**Figure 3:**
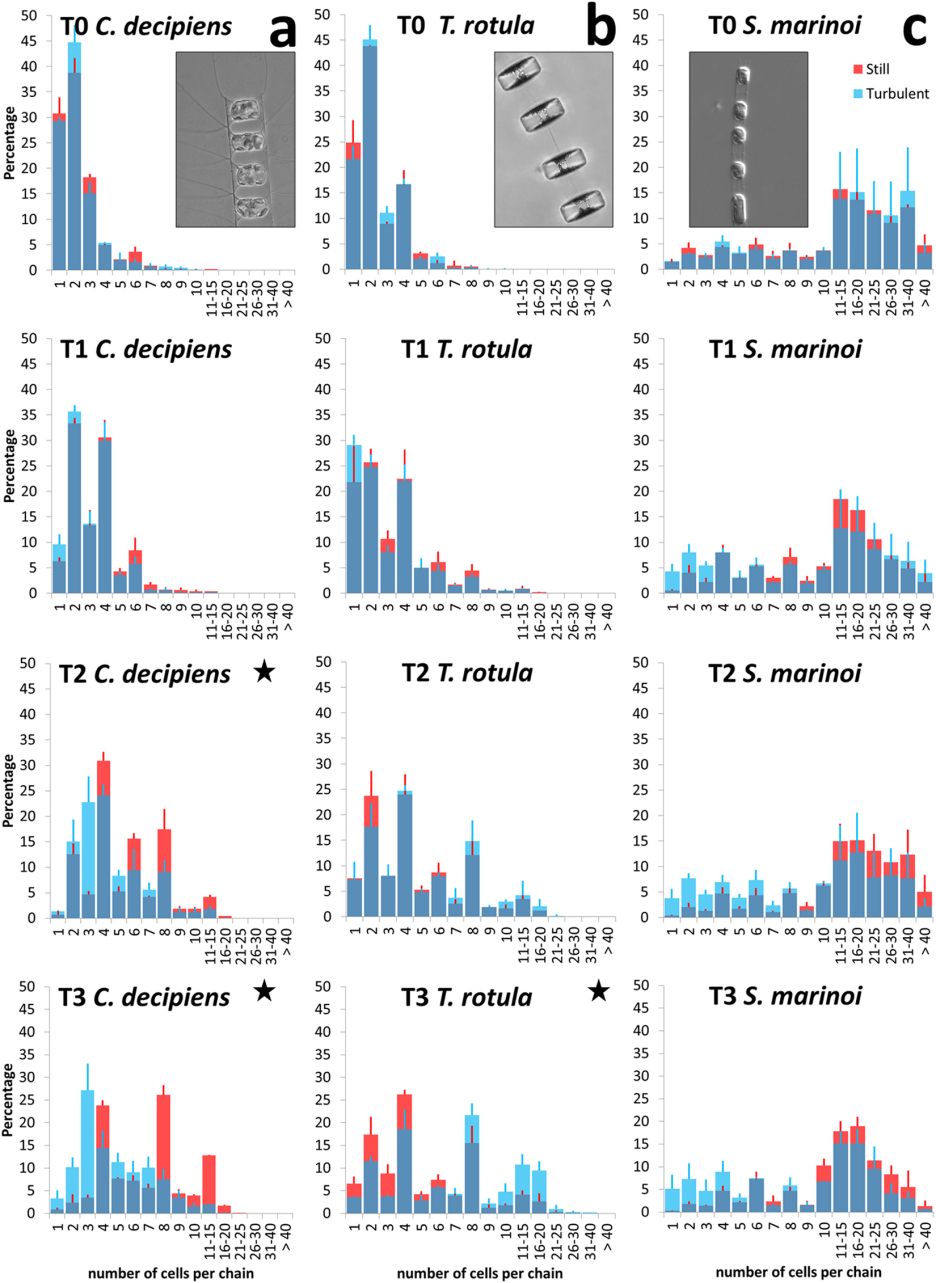
Chain spectra over time (T0-T3) in the three tested species. (**a**) *Chaetoceros decipiens*, (**b**) *Thalassiosira rotula*, (**c**) *Skeletonema marinoi*. A representative chain of each species is depicted in the inlets (courtesy of I. Percopo). On the x-axis the chain length expressed as number of cells per chain is reported, on the y-axis the frequency of each chain class expressed in percentage. Dark blue portions of the histograms indicate overlap, red histograms indicate enrichment of a given chain class in the still condition, blue histograms indicate enrichment in the turbulent condition. The reported values are average of three replicates; vertical bars indicate standard deviations (red for still and blue for turbulent conditions, respectively). All results were statistically validated by Kolmogorov-Smirnov two-sample (KS2) and Wilcoxon non parametric tests (Supplementary Tables 8 and 9), a black star indicates that test outcome equals 1.

**Figure 4:**
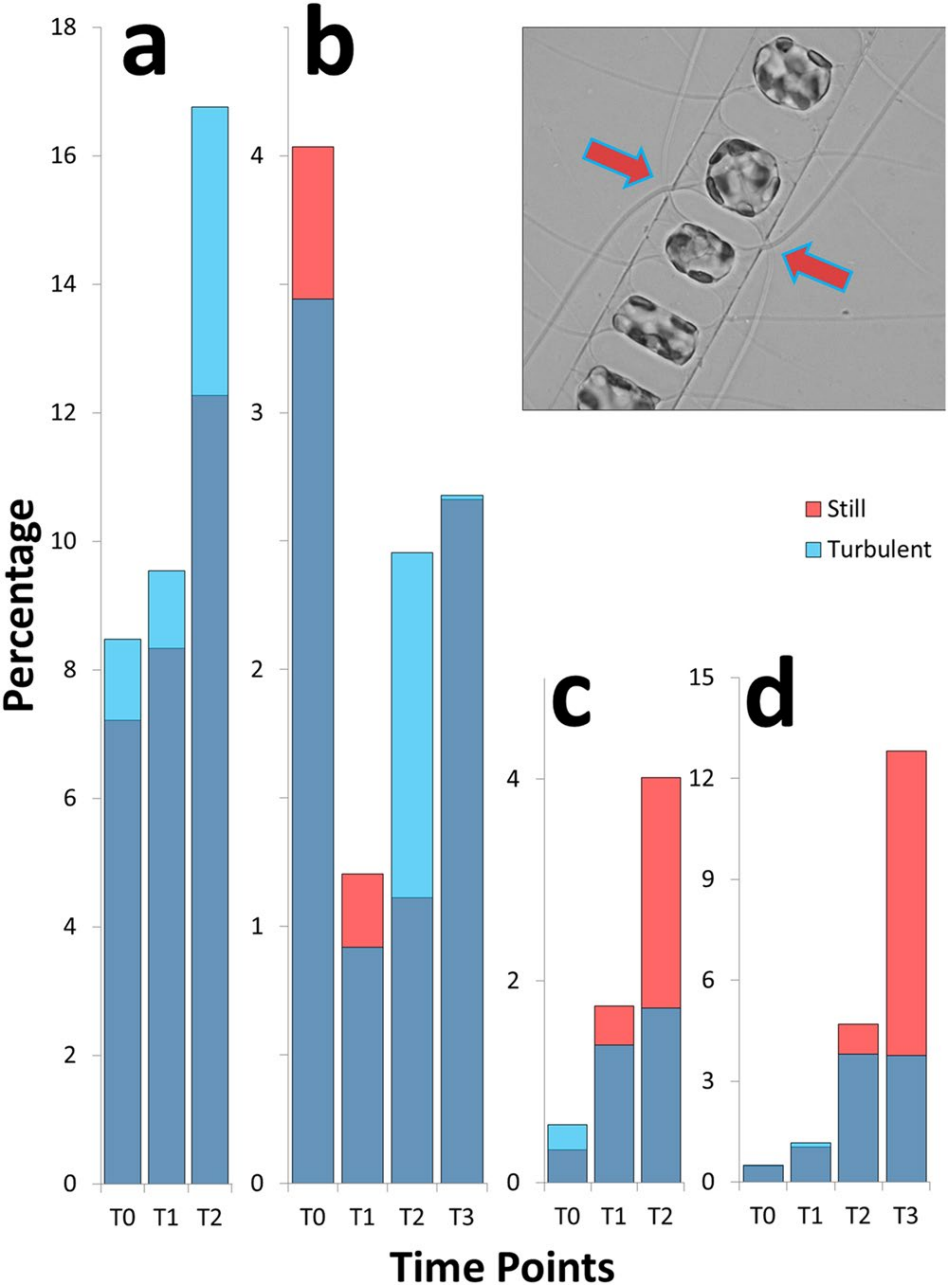
Frequency of separating chains (**a**,**b**) and mechanically broken chains (**c**,**d**) in *C. decipiens*. (**a**,**c**) experiment 1; (**b**,**d**) experiment 2. On the x-axis time points are reported, on the y-axis the frequency of separating (**a**,**b**) and mechanically broken (**c**,**d**) chains expressed as percentage are reported. Colour code follows Fig. 3. Inlet: a separation point with two adjacent separating cells. Arrows indicate thicker terminal setae produced by the separating cells.

In *C. decipiens*, the metabolic response depicted above was accompanied by a morphological modification in the average chain length (Fig. 2e,f) and chain spectra; namely shorter chain classes resulted significantly enriched in turbulent condition (Fig. 3a and Supplementary Tables 8 and 9), in accordance with the observed reduction of the average chain length. One of the interesting features characterising *C. decipiens* is that broken chains can be easily recognised in this species because, when a chain mechanically breaks, the apices of the resulting chains will lack apical cells discernible by their thicker setae spanning parallel to the chain length. In our experiments the overall incidence of broken chains was ∼1% and ∼2% in turbulent and still conditions respectively for experiment 1 and ∼3% and ∼6% for experiment 2 (Fig. 4c,d; Supplementary Table 10). The frequency was higher in still conditions where no grids passed through fluid and disturbed cells (Fig. 4c,d). When a *C. decipiens* chain naturally splits into two or more subchains upon mitosis, a so-called separation point appears. A separation point is characterised by two adjacent separating cells that synthesise apical setae instead of intercalary setae (Fig. 4, inlet). These will become apical cells of the new subchains deriving from the separation. The number of chains that presented at least one separation point was higher in turbulence than in still condition at T2 (Fig. 4a,b) thus *C. decipiens* chains were more prone to separation.

The reason for such a response is not clear, but that chain length regulation is the most evident read-out for diatoms exposed to turbulence was validated by the experiments carried out on *T. rotula* and *S. marinoi* (Fig. 2g,h). The three diatom species used in our experiments responded differently to the turbulent stimulus applied. *T. rotula* exposed to turbulence responded producing significantly (Supplementary Tables 8 and 9) longer chains (Fig. 3b and Supplementary Fig. 3c). For *S. marinoi* the response was not statistically significant (Fig. 3c and Supplementary Table 8). Internal controls, i.e. statistical tests run between replicates (e.g. turbulent cylinder A vs turbulent cylinder C) showed that from time point T1 onward the replicates were not statistically similar among each other (Supplementary Table 8). If the accuracy level was reduced from 99.9% to 95%, internal controls resulted consistent with statistically comparable replicates at T0 and T1 but not at T2 and T3. Mercurialness of replicates can be due to intrinsic variability of the species. Nevertheless, the mean chain length was slightly reduced in presence of turbulence as compared to still condition (Fig. 2h) although the standard deviations overlapped.

## Discussion

One of the tenets in biological oceanography is that non-motile organisms below a certain threshold scale are not able to perceive turbulence^2, 3, 41^. Consequently diatoms, incapable of active movements, are universally believed to do better in turbulent waters only because turbulence mobilises nutrients and distorts the boundary layer around the cells, enhancing nutrient gradients towards the cell wall and therefore uptake. This implies that in nutrient repletion (i.e. when nutrients are never limiting for growth) diatoms should not be affected by turbulence. Previous studies have demonstrated that diatoms are favoured by turbulence in mesocosm experiments^7, 9, 42, 43^ and *in situ* ^44^, but how the response is triggered is unclear.

We compared morphology and gene expression profile of *Chaetoceros decipiens* cultures exposed to natural levels^27^ of microscale turbulence to cultures kept in still condition. We believe that turbulence is the main factor these responses can be ascribed to, because all the other growth parameters were kept constant in the two sets of triplicates, including nutrient concentrations and light supply. In nutrient replete condition, the medium concentrations of the three main nutrients (i.e. nitrates, phosphates and silicates) could sustain a flux toward the cell wall in still water from seven to eight orders of magnitude larger than the uptake rate, thus preventing any diffusion limitation in still water. This means that the boundary layer around the cells is never depleted of nutrients and therefore turbulence would not affect nutrient uptake and consumption. Light intensity cannot be a driver for the observed changes either. Considering that the cell concentrations in all cylinders were comparable, there was no effect of light attenuation. We also considered that cells in turbulence experienced a fluctuating light regime with respect to those in still^45^ but since the illumination was from the side and the cylinder radius was 0.12 m^28^, the maximum light variation between the wall and the centre of the cylinder was less than 5%, and overall fluctuation in the 3D space of the cylinder accounted for no more than 13%, even at the maximum cell concentration recorded.

Turbulence should not be a stressor like nutrient depletion or predators^46, 47^ nor a strong source of signals like light can be for photosynthetic organisms^19, 21^, still the cell response after 48 hours from the onset of turbulence was remarkable, with ∼12% of the total *C. decipiens* transcripts differentially expressed. The response was less strong at T3, with ∼2% of differentially expressed transcripts, and this could reflect an adaptation or a reduced sensitivity to the imposed turbulent regime, suggesting that the strongest changes occur in the first 48 hours after turbulence is applied. Cell-to-cell communication is likely occurring in a chain to regulate its length. The transcriptional changes related to this communication would be hard to capture with transcriptomics as i) the portion of the population that responds to turbulence by tuning chain spectrum is small and the transcriptional signal would be diluted in the background noise; ii) cells may communicate also in still condition^29^.

In the first reported evidence that a diatom, *Phaeodactylum tricornutum*, could sense and respond to a mechanical stimulus, the response was followed by measuring calcium waves within seconds after the application of the stimulus^17^. In our DE analyses different enzymes catalysing phosphorylation of phosphatidylinositol were variably regulated at T2. These molecules are involved in G protein signalling pathway and in cytoplasmic calcium release to trigger a cascade of protein modifications that lead to a cellular response. Further functional studies would be required to clarify if in *C. decipiens* G proteins are involved in turbulence perception. NDPKs as well are involved in signal transduction and in our experiments three transcripts were regulated. This can be a starting point for further investigations, which will examine earlier phases of the response.

Gene expression changes on the other hand indicate more clearly that metabolic changes are occurring, most likely in the entire cell population. Fatty acid (FA) biosynthesis was one of the significantly enriched pathways among DE genes (Fig. 1b,c, adjusted p-value 0.007). FA can be employed in at least three different ways in a cell^48^, i.e. as cell-to-cell signalling molecules, as storage compounds or to produce cell membranes. A lipid content increase was measured and correlated to Si-NaCl double deprivation stress^49^ or to culture senescence^50^ in a congeneric species (*Chaetoceros gracilis*) and FA in those experiments represented up to 93% of the total lipid cellular content. Also in *Thalassiosira pseudonana* FA accumulation was related to nutrient stress^51^. In our case, cells did not experience any nutrient stress, as confirmed by data from a similar experiment^52^.

The up-regulation of FA biosynthesis was recorded when *P. tricornutum* cells experienced the transition from dark to light^53^. When light turns on, cells start fixing carbon very intensively and possibly the excess of energy produced is deviated to the synthesis of FA that can be turned into triacylglycerols (TAG) for storage as oil droplets^54^. Similarly to what reported in *P. tricornutum* when light turns on^53^, also in *C. decipiens* under turbulent conditions some desaturase-encoding genes^55^ were up-regulated together with an elongase. In *P. tricornutum*, reactivation of photosynthesis after a period of inactivity induces cells to redirect the surplus of fixed carbon to FA production^53^; in turbulence *C. decipiens* showed a similar transcriptional response. As mentioned above, the fate of the FA synthesised can be storage, or they can be used for membrane polar glycerolipids. FA can also serve as substrate for oxylipin production^56^, compounds that play a role as signalling molecules. Isoprenoids are important compounds among which sterols, hormones, and carotenoids are the most known. We observed an over-expression of the PPP that would increase the GAP content in the cells that can be used to produce precursors for sterols. These compounds are known to serve a function as membrane signal transducers^57, 58^ and possible involvement of sterols in *C. decipiens* response to turbulence is corroborated by the up-regulation of a transcript containing squalene cyclase domains (c9513, Supplementary Table 1). Squalene cyclase is involved in the first crucial steps of all sterol biosynthesis, i.e. cyclisation of linear precursors^57^.

Besides annotated genes, a relevant number of unknown transcripts showed regulation in turbulent vs. still condition. This very high rate of unknown DE transcripts can be an indication of uniqueness of these fascinating algae, characterised by a peculiar chimeric genome^59^ or of very peculiar responses that diatoms carry out to cope with fluid motion.

We cannot exclude that the differences in expression patterns between the two treatments may partly reflect the weighted average of responses of cells in still conditions which were experiencing a larger inhomogeneity in growth conditions on the time scale of a day.

Specifically, small differences in division rates and inhomogeneous exposure to light might contribute to, but certainly not entirely account for, the response. We believe that none of these factors on its own is relevant enough to justify the changes and, more importantly, that nutrient fluxes and light fluctuations, the two processes so far considered as mostly affected by turbulence, might not be considered the drivers of the observed response.

The transcriptional response was accompanied in *C. decipiens* with a morphological modification and a one-day lag phase of growth. The latter can be due to the metabolic switch from active growth to accumulation. And in fact the same lag-phase was observed in *T. rotula* cultures exposed to turbulence but not in still *T. rotula* cells. The three species examined here are characterised by different junction modes, chain shape and stiffness^60, 61^, and also different cell sizes. These three species presumably interact differently with the mechanical movements of the fluid^13^.

Possible scenarios to explain how diatoms sense turbulence can be advocated; fluid movement causes a pressure wave that is perceived on the cell wall via transmembrane proteins that change conformation and send a signal to the cell interior. Membrane curvature sensing (MCS) mechanism^62^ could be hypothesised^63^, with SYLF domain containing proteins involved^64^. In our differential expression analysis the transcript c9791 contains a SYLF domain and results slightly up-regulated in turbulent condition (Supplementary Table 1).

Further investigations are needed to decipher molecular and physiological mechanisms devoted to turbulence sensing in diatoms, but the intriguing question that can have profound ecological implications is why do diatoms respond to turbulence ? For a diatom, incapable of active movements, turbulence means mixing and mixing can lead to a displacement out of the euphotic zone. A possible scenario would be that if diatoms can promptly cope with a period in the dark relying on the FA accumulated then they can survive to a passage out of the euphotic zone. Once in the dark they can convert FA to energy and wait for turbulence to bring them back in the upper layers. FA may also serve as buoyancy regulators^65^ and their accumulation upon turbulence perception can be interpreted as a way to balance the movement toward the deep dark ocean.

## Conclusions

The results presented here suggest that diatoms sense turbulence in nutrient repletion conditions and respond to it by varying chain spectra and presumably metabolic state, suggested by gene expression changes. This was completely unexpected based on previous physical and biological knowledge^5, 11, 14, 66^. Our observations on chain spectra and separating chains in turbulence vs. still conditions testify the existence of a mechanism to perceive fluid movements. The present work showed that diatoms not only perceive and respond to mechanical stimuli within seconds from the application of the stimulus^17^, but also respond morphologically as well as transcriptionally in the first days after turbulence is applied. Furthermore, different marine centric diatom species showed individual responses to the same stimulus. Understanding how this can shape the lower levels of the oceanic food web can shed light on the possible evolution of marine ecosystems in response to physical environment.

## Methods

### Strains and experimental plan

The non-axenic *Chaetoceros decipiens* SZN-Cdec and *Thalassiosira rotula* SZN-Trot cultures were obtained by single chain isolation from net samples. The non-axenic *Skeletonema marinoi* strain V32 was a kind gift of Dr. Anna Godhe (University of Gothenburg, Sweden). Cultures were grown in F/2 medium^67^. Temperature for culture maintenance was set at 18 ± 1 °C, photoperiod at 12 L:12D and irradiance at 80 µmol photons·m^−2^·s^−1^. Experiments were run using TURBOGEN^28^, a prototypic instrument composed of six 13-L cylinders for algal growth. Supplementary Fig. 1 depicts the workflow of the experiments run in the present investigation.

### *Chaetoceros decipiens* experiments

Two experiments, named ‘experiment 1’ and ‘experiment 2’, were carried out with *Chaetoceros decipiens* strain (cell diameter ∼23 µm). The strain was kept in active and exponential growth phase for two weeks before the experiment by serial dilutions. One week before the experiment a 5 L Pyrex^®^ sterile Erlenmeyer flask was inoculated and the growth was followed for 6 days. At the moment of the inoculum of the TURBOGEN cylinders for experiment 1, a cell count was carried out by Sedgwick-Rafter counting chamber^68^ in an inverted light microscope at 400× magnification and the cell concentration estimated at 1.3 × 10^4^ cells·ml^−1^. Cells were in exponential growth phase. The total amount of F/2 medium needed for the experiment (13 L per cylinder × 6 = 78 L) was inoculated with 1.95 × 10^7^ cells to get to a final concentration of 250 cells·ml^−1^. Five hundred ml of the culture used for the inoculum were diluted in the same 5 L Pyrex^®^ Erlenmeyer flask to be used for the next experiment. After one week, during which experiment 1 was carried out, cells in the Erlenmeyer flask were counted to be used as inoculum for experiment 2; the cell concentration was estimated at 1.5 × 10^4^ cells·ml^−1^. 1.95 × 10^7^ cells were then inoculated as before to get to a final cell concentration of 250 cells·ml^−1^. For both experiments, the 78 L were then distributed over the six cylinders composing the TURBOGEN (time point T-1) and left 24 hours for acclimation after inoculum. Temperature, photoperiod and light intensity in the TURBOGEN were set as for maintenance. After 24 hours from the inoculum (time point T0), all the cylinders were very gently stirred for 15 seconds (∼60 r.p.m.) with a 25-ml-strippette (Corning^®^ Costar^®^ Stripette^®^, cat. n. 4490, Corning Incorporated NY 14831, USA) in order to homogenise the cell suspension. A 30-ml-sample was then taken from each cylinder and fixed with neutralised formaldehyde (final concentration 1% v/v) and stored at 4 °C in the dark until analysis. At T0 vertically oscillating grids were mounted in TURBOGEN and turbulence was activated in three out of the six cylinders (namely cylinders A, C, and E) with the following settings: stroke 240 mm, grid speed 100 mm·s^−1^, acceleration 1000 mm·s^−2^. The turbulent kinetic energy dissipation rate, ε, was in the order of 10^–4^ m^2^·s^−3^, i.e. in a range where nutrient fluxes around cells below 30 µm are not enhanced by turbulent motion^14^. In cylinders B, D, and F no turbulence was imposed in order to have a negative control condition referred to as ‘still condition’. Experiment 1 lasted 3 days (from time point T0 to T2; i.e. 48 hours of continuous turbulence), while experiment 2 lasted 4 days (from time point T0 to T3; i.e. 72 hours of continuous turbulence). At time point T2 for experiment 1 and time point T3 for experiment 2, cells were collected by filtration for RNA isolation and sequencing. Every day at the same time stirring and sampling were carried out as described above. The turbulent cylinders were not stirred. In pilot experiments, cells in still cultures in two cylinders were counted before and after stirring, to verify that no sinking occurred, and we obtained the following values: cylinder 1 at T2, 1635 cells·ml^−1^ before and 1609 cells·ml^−1^ after stirring, cylinder 2 at T2, 1305 cells·ml^−1^ before and 1255 cells·ml^−1^ after stirring; cylinder 1 at T3, 3311 cells·ml^−1^ before and 3710 cells·ml^−1^ after stirring, cylinder 2 at T3, 2733 cells·ml^−1^ before and 2776 cells·ml^−1^ after stirring.

### *Thalassiosira rotula* and *Skeletonema marinoi* experiments

The experiment with *T. rotula* and *S. marinoi* were conducted exactly like what reported above for *C. decipiens* (Supplementary Fig. 1). At the moment of the inoculum of the cylinders, cell concentrations were estimated at 7.6 × 10^3^ and 9.8 × 10^4^ cells·ml^−1^ for *T. rotula* and *S. marinoi* respectively. Cells were in exponential growth phase. The total amount of filtered F/2 medium (78 L) was inoculated with 3.9 × 10^7^ and 1.95 × 10^8^ cells to get to a final concentration of 500 and 2.5 × 10^3^ cells·ml^−1^ for *T. rotula* and *S. marinoi* respectively.

### RNA-seq and qPCR

For *C. decipiens* experiments cells were gathered by filtration and RNA isolated by Roche TriPure® reagent following manufacturer’s instructions. RNA samples were analysed at an Agilent 2100 Bioanalyzer platform to assess integrity, at a NanoDrop 2000 Spectrophotometer to assess purity, and quantified at a Qubit fluorometer. 1.5 µg RNA from two still and two turbulent samples from each time point were sent to EMBL Gencore Facility for sequencing on the Illumina HiSeq2000 platform; single end 50 bp reads were produced. RNA-seq data are available in ArrayExpress under accession number E-MTAB-5031.

1 µg RNA from three turbulent and three still condition samples from each time point was reverse transcribed using Qiagen QuantiTect Reverse Transcription Kit following manufacturer’s instructions for qPCR validation of RNA-seq results.

### Cell counts and data analyses

Cell counts were used to define chain distribution over time as well as to draw growth curves. On average 427 chains per sample were counted in Sedgwick-Rafter or Utermöhl^69^ counting chambers. Statistical relevance of results was estimated by applying Wilcoxon and Kolmogorov-Smirnov two sample (KS2) tests (99.9% accuracy, α 0.001) in MatLab.

### Bioinformatics analyses

The fastq files from RNA-seq experiments were inspected using FastQC tool and further cleaned and trimmed using Trimmomatic. Trinity software (ver. trinity_201407) was used for the assembling of the reads. We quantified transcript expression levels by mapping reads against the assembled transcriptome using Bowtie (ver. 1.1). To count the reads mapped we used Samtools (ver. 0.1.19-44428 cd). The resulting reference transcriptome was annotated using Annocript^70^ software that aligns transcripts against known proteins, domains and non-coding RNAs (Supplementary Information). In order to refine domain annotation we used META-CLADE (Ugarte *et al*., unpublished), a software designed on the basis of CLADE^71^ with the purpose to annotate metagenomics and metatranscriptomics reads. META-CLADE exploits multiple probabilistic models representing protein domains and characterising different evolutionary pathways. Multiple models have been constructed for all known Pfam domains. META-CLADE combines this multiple source annotation strategy with domain based learned estimators of domain annotation. We mapped Pfam domains into contig sequences, annotated the contigs and made the analysis (counting) on each sample. The importance of a functional class is highlighted by the abundance of the domains within the class. To compare the estimations obtained on each sample, we normalised the abundance with respect to the size of each sample (annotation per megabase).

### Differential expression analysis

We used EdgeR to select transcripts differentially expressed (DE) between still and turbulent conditions. Transcripts were considered as DE if the false discovery rate (FDR) was smaller or equal to 0.05 and the fold change greater than 2. Enrichment analyses for GO terms and Pathways were performed exploiting the Fisher exact test and the Benjamini and Hochberg correction of the p-values. GO terms and Pathways were considered enriched when associated to at least 10 DE transcripts with an adjusted p-value smaller than 0.1.

Supplementary Information contains detailed descriptions of all the methods reported in the present section.

## Electronic supplementary material

Supplementary Information

Supplementary Table 1

Supplementary Table 2

Supplementary Table 11

Supplementary Table 12

Chaetoceros decipiens de novo transcriptome
