# Supplementary material for: Marine diatoms change their gene expression profile when exposed to microscale turbulence under nutrient replete conditions
Source: Sci Rep. 2017 Jun 19;7:3826. doi: 10.1038/s41598-017-03741-6 (PMC5476593; doi:10.1038/s41598-017-03741-6)
Supplement: Supplementary file 1 — Supplementary Information [file 41598_2017_3741_MOESM1_ESM.pdf]

Marine diatoms change their gene expression profile when exposed to microscale turbulence under nutrient replete conditions

Alberto Amato, Gianluca Dell'Aquila, Francesco Musacchia, Rossella Annunziata, Ari Ugarte, Nicolas Maillet, Alessandra Carbone, Maurizio Ribera d'Alcalà, Remo Sanges, Daniele Iudicone, Maria I. Ferrante

Supplementary Methods

Cultivation details and medium preparation. The non-axenic *Chaetoceros decipiens* SZN-Cdec and *Thalassiosira rotula* SZN-Trot cultures used in this paper were obtained by single cell or single chain isolation from net samples collected at the long term monitoring station MareChiara (LTMS MC[1]). The non-axenic *Skeletonema marinoi* strain V32 was a kind gift of Dr. Anna Gohde (University of Gothenburg, Sweden). Isolation was performed using a flame-capillarised glass Pasteur pipette using a Leica light microscope at a 100x magnification. Temperature was set at 18 ± 1°C, photoperiod at 12L:12D and irradiance at 80 μmol photons·m^{-2}·s^{-1}. F2 medium[2] was prepared by adding the adequate quantity of nutrients from single solutions following Guillard's recipe[2] to ultrafiltered, autoclaved sea water collected at the LTMS MC. Superficial sea water samples were first filtered through Millipore 0.22 μm membrane filters (Millipore nitrocellulose filter, GSWP09000, Merck Millipore Darmstadt, Germany) then all the nutrients but vitamins were added. The medium was then left stand for 24 hours to let the chemical species reach equilibrium. The medium was filtered through 0.45 μm Millipore membranes (Millipore, HAWP09000) to remove flocculates that form after nutrients (especially $Na2SiO_3 \cdot 9H_2O$) addition. Autoclave sterilization for 1 hour at 121°C in 10L-Nalgene polycarbonate bottles was performed. Once the temperature reduced to less than 37°C, vitamins were added under sterility conditions. The medium needed for the experiments was stored for at least 48 hours in the growth chamber where the experiments were carried out in order to have the medium at the same temperature of the chamber and thus avoid thermal shock to the cells at the moment of the inoculum. The sea water to prepare culture medium for *S. marinoi* was lowered in salinity to 26 psu by adding MilliQ water because the strain was isolated from the Northern Sea where the salinity is lower compared to LTMS MC.

Experimental set-up. TURBOGEN[3] is a prototypic instrument designed and produced in collaboration with M2M Engineering. It is composed of six 13L Plexiglass® cylinders. A fully digitally controlled high performance engine moves circular grids into the cylinders in order to produce turbulence. At the conditions imposed for our experiments (stroke 240 mm, grid speed 100 mm·s^{-1}, acceleration 1000 mm·s^{-2}), the turbulent kinetic energy dissipation rate, ε, was in the order of 10^{-4} m^2·s^{-3}, i.e. in a range where for cells of the size of those used here the flux of nutrients toward the cell is not enhanced by turbulent motion.
For an extensive technical overview of the instrument and the calibration procedure and results see ref. [3].

RNA isolation, reverse transcription and qPCR. For experiments 1 and 2, 1 to 1.5x10^7 cells were filtered onto 1.2 μm-porosity Millipore RAWP membrane filters (Mf-Millipore RAWP04700). Membrane filters were mounted onto filter holders then washed with sterile

MilliQ water. After filtration, 50 ml of PBS were used to wash the filters. Filters were cut into two halves with a razor blade and put into 2 ml Eppendorf tubes previously filled with 1.5 ml of Roche TriPure® (Sigma - Aldrich s.r.l. Milan, Italy, cat. n. 011667157001) isolation reagent. Eppendorf tubes were snap-frozen in liquid nitrogen. Samples were stored at -80°C until use. RNA was isolated from TriPure® frozen filters with the following protocol. TriPure®-soaked half filters were thawed at room temperature. A dash of Sigma 212-300 µm acid-washed glass beads (Sigma, cat. n. G1277) were added and incubated for 10 minutes at 60°C on a thermoshaker at 1,400 rpm. Tubes were spun to get rid of the beads and the filter. The supernatant was transferred to a new 2-ml Eppendorf tube and 200µl of chloroform per ml of TriPure® were added. Tubes were vigorously shaken for 15 s and incubated for 15 minutes at room temperature. Tubes were centrifuged at 10,600 rpm (12,000 g) for 15 minutes at 4°C. The upper aqueous phase containing RNA was transferred to a new 2 ml Eppendorf tube and an equal volume of isopropanol added to precipitate RNA. Tubes were gently mixed by inversion and incubated at room temperature for 10 minutes. Samples were centrifuged at 10,600 rpm for 10 minutes at 4°C to pellet down RNA salts. One ml of 75% ethanol prepared with DEPC-treated water was used to clean the RNA pellet. Tubes were centrifuged at 8,500 rpm (7,500 g) for 10 minutes at 4°C, the ethanol discarded and let the pellet air-dry for 20-30 minutes. The RNA pellet was resuspended in 20-30 µl of RNAse-free water and incubated for 5-10 minutes at 55°C to facilitate elution. RNA samples were DNAse-treated (Roche DNase I recombinant, RNase-free, cat. n. 04716728001) to get rid of genomic DNA contamination and cleaned up by Qiagen RNeasy Mini Kit (Qiagen, Hilden, Germany, cat. n. 74106). Samples were stored at -80°C until use.
RNA samples were analysed at an Agilent 2100 Bioanalyzer platform (Agilent Technologies 5301 Stevens Creek Blvd. Santa Clara, California 95051 USA) to assess integrity, at a NanoDrop 2000 Spectrophotometer (Thermo Fisher Scientific Inc., Waltham, Massachusetts, USA) to assess purity, and quantified at a Qubit fluorometer (Thermo Fisher Scientific Inc.) following manufacturer's instructions. 1.5 µg RNA from two still and two turbulent samples from time points T2 (experiment 1) and T3 (experiment 2) were sent to EMBL Gencore Facility for sequencing at the Illumina HiSeq2000 platform, producing non paired-end 50 bp reads.
1 µg RNA from three turbulent and three still samples from time point T2 (experiment 1) and T3 (experiment 2) was reverse transcribed using Qiagen QuantiTect Reverse Transcription Kit (cat. n. 205313, Qiagen) following manufacturer's instructions for qPCR validation of RNA-seq results. Primers used in this paper are listed in Supplementary Table 4. For qPCR 1 µl of a 1:5 dilution was used in each 10 µl reaction. The reaction contained: 5 µl Fast SYBR® Green Master Mix (Thermo Fisher Scientific Inc., cat. n. 4385612), 7 pmols of each primer and 1 µl of 1:5 cDNA dilution. An Applied Biosystems ViiA™ 7 Real-Time PCR System (Thermo Fisher Scientific Inc.) was used with the following reaction programme: a first denaturation step at 95°C for 20 s then 40 cycles at 95°C for 1 s, 60°C for 20 s. For melting curve production the following cycle was run after the amplification: 95°C for 15s, 60°C for 60s and 95°C for 15 s.

Cell counts and data analyses. Cell counts were used both to define chain distribution over time and to draw growth curves. On average 427 chains per sample (16,817 total chains for *C. decipiens* over a total of 42 samples counted; 10,538 for *T. rotula* and 10656 for *S. marinoi* over a total of 24 samples per species) were counted in Sedgwick-Rafter counting chambers in a light microscope at a magnification of 100x. For *S. marinoi*, Utermöhl[4] counting chambers were used instead, at an inverted microscope (magnification 200x). For chain distribution every chain class was weighed on the total number of chains counted and expressed in percentage. The division rate was calculated as the slope of the growth curve

built with log_2-transformed cell density values between every time point both in single cylinders and on the mean values. The maximum division rate was calculated on the steepest portion of the growth curve. For *C. decipiens* the number of separating chains (chains presenting at least one separation point identified by two adjacent separating cells within the chain itself, see Fig. 3, inlet) was recorded as well. The chain length of the separated subchains was recorded and compared among replicates and between treatments and controls. The number of mechanically broken chains was recorded as well. Broken chains can be recognised because at one or both apices they lack the terminal cells, identifiable by the presence of terminal setae (see main text and Fig. 2, inlet).
Statistical relevance of results was estimated by applying Wilcoxon and Kolmogorov-Smirnov two-sample (KS2) tests (alpha 0.001 i.e. 99.9% accuracy) in MatLab. If the null hypothesis (samples are identical) can be rejected, the test result equals 1, otherwise the outcome equals 0.
Both tests were carried out between replicates (turbulent vs. turbulent or still vs. still) in order to verify congruence of replicates and between experimental and control samples (turbulent vs. still). All possible pairwise combinations were tested.

Generating a reference transcriptome. Sequencing of the cDNA from the *Chaetoceros decipiens* samples was performed using Illumina technology. The resulting fastq reads files were inspected using FastQC tool (http://bit.ly/1aNGclw) and further cleaned from adapters and trimmed for quality using Trimmomatic with the following parameters: ILLUMINACLIP: TruSeq3-SE.fa:2:30:10:1:true SLIDINGWINDOW:3:28 MINLEN:40[5]. Trinity software (ver. trinity_201407) was used for the assembling of the reads using these parameters: --normalize_reads --inchworm_cpu 24 --bflyHeapSpaceInit 24G --bflyHeapSpaceMax 240G --bflyCalculateCPU --CPU 24 --jaccard_clip --min_kmer_cov 2. Here Jaccard clip was used to mitigate false fusion of adjacent transcripts[6]. The assembling resulted in 27,923 contigs with a N50 value of 1,912 bp and average contigs length 1,211 bp. We chose to filter out transcripts showing no relevant expression. To this aim, we quantified transcript expression levels by mapping reads against the assembled transcriptome using Bowtie (ver. 1.1) with parameters: --p 20 --chunkmbs 10240 --maxins 500 --seedlen 20 --tryhard -a –S[7]. To count the reads mapped we used the idxstats program in Samtools (ver. 0.1.19-44428cd). Only transcripts with count per million (CPM) greater than one for at least 2 out of 8 samples were considered expressed beyond background noise and retained.

Transcriptome annotation. The resulting reference transcriptome contains 21,224 transcripts which were annotated using the software Annocript[8] (Supplementary Table 12). This pipeline permitted to align the transcripts against known proteins, domains and non-coding RNAs. A blastx[9] against SwissProt[10] and UniRef90[11] (version 2014_08) was performed using the following parameters (-word_size 4 -evalue 1E-5 -num_threads 20 -num_descriptions 5 -num_alignments 5 -threshold 18). The rpstblastn against the Conserved Domains Database (CDD) returned the domains composition of the putative proteins using these parameters: -evalue 0.00001 -num_descriptions 20 -num_alignments 20. The CDD database contains domains models from Pfam, SMART, COG, PRK and TIGRFAM[12]. Blastn against Rfam (performed with parameters: evalue 0.00001 -num_threads 4 -num_descriptions 1 -num_alignments 1) permitted the annotation of putative non-coding ribosomal RNAs[13]. Moreover, the best scored proteins in Annocript are associated with Gene Ontology (GO) terms[14], enzymes from the ExPASy database[15] and UniPathways[16]. The specific databases used can be found at the Annocript GitHub reference page (http://bit.ly/1y6Ixoo).

Refinement of domain annotation by Meta-CLADE. The importance of a fine domain annotation is crucial to estimate the abundance of certain functional classes of domains within the community and the absence of others, and to highlight enzymatic biochemical functions associated to specific environmental substrates. Probabilistic models generated from the consensus of a set of homologous sequences are often used as representations of protein domains. When sequences are well conserved, the resulting probabilistic model captures the most conserved features in the domain sequences and these latter can be successfully used to find new domains in databases of sequences, sharing the same features of the original sequence. However, when sequences have highly diverged, consensus signals become too weak to generate a useful probabilistic representation, and models constructed by global consensus do not properly characterize domain features. To overcome this fundamental bottleneck, we introduced CLADE-centered models[19], defined by considering a set of homologous sequences, and we constructed a model for each sequence Si from a set of conserved sequences that are similar to Si. By so doing, the probabilistic models generated to represent a domain, are displaying features that are characteristic of each sequence Si, and they might be very different from Si to Sj. More divergent the homologous domain sequences Si and Sj are, more the models constructed from these sequences are expected to display different features. It is therefore important for a domain to be represented by several models that can characterise its different paths of evolution within different clades. The multi-source representation has been demonstrated to be more efficient than a single source strategy for domain identification in Bernardes et al.[19]. Based on multiple models associated to each domain, we constructed a large probabilistic model library for all Pfam domains. For each domain, the library includes the Pfam consensus models and about 350 clade-centered models. This amounts to more than 2.5 million probabilistic models. Such models have been previously used in CLADE[19], a tool for domain identification designed for improving genome annotation. META-CLADE (Ugarte et al, in preparation) has been designed based on CLADE with the purpose to annotate metagenomics and metatranscriptomics reads. Due to the read features described above, these data demand a special treatment and a specific algorithmic design. META-CLADE is based on two main steps. The META-CLADE first step takes as input a set of metaG/metaT reads or contigs where CDS/ORFs have been previously annotated, together with all probabilistic CLADE-centered models and Pfam pHMM models. For each sequence, the CDS region is scanned with the probabilistic models and all domain hits are identified. For each hit, two scores are assigned, the bit-score for the entire hit and the mean-bit-score for each hit residue (obtained as the bit-score of the hit divided by the length of the hit). The META-CLADE second step estimates domain specific thresholds for bit-scores and mean-bit-scores, that best separate true hits from false hits, for each domain. Hence, each CDS sequence is represented by a set of domain hits, where each domain hit is described by its bit-score, mean-bit-score, predicted state (positive or negative) and probability of being a positive/negative prediction. The bit-score and mean-bit-score are provided by the matching of a probabilistic model, and the predictive state of the hit together with its probability are provided by a learning step. Then, the set of hits is filtered in several steps: 1. all pairs of overlapping hits that are associated to the same domain and that cover at least 85% of the length of at least one of the two hits, are filtered from the list of CDSs. For such pair of hits, we eliminate the hit that has the lower bit-score. 2. Second, META-CLADE filters out all hits having a bit-score which is smaller than the threshold provided by positive sequences, as determined in the analysis of fragmented segments in read sequences. 3. Based on the parameter estimation obtained with the BayesNaiveBayes method applied to each Pfam domain, the filter accepts only those domain hits that are positive hits and whose probability p for being a positive hit is $p > .9$. 4. Hits are filtered by best E-value score, associated to each CLADE-centered model and pHMM model. Namely, domain hits are

ordered by considering lowest E-value scores and iteratively eliminated hits whether they overlap some domain at least 15% of the length of the hit sequence having best E-value. The output of this filtering pipeline is the contig annotated with non-overlapping domain hits.

Differential expression analysis. A plugin, using EdgeR libraries[17] is included in Annocript and is used to select transcripts that are differentially expressed between still and turbulent conditions. Transcripts were considered differentially expressed if the false discovery rate (FDR) was smaller or equal to 0.05 and the fold change greater than 2. Another included plugin was used to perform enrichment analysis of GO terms and Pathways exploiting the Fisher exact test and the Benjamini and Hochberg correction of the p-values. GO terms and Pathways were considered enriched when associated to at least 10 differentially expressed transcripts with an adjusted p-value smaller than 0.1.

Supplementary Figures

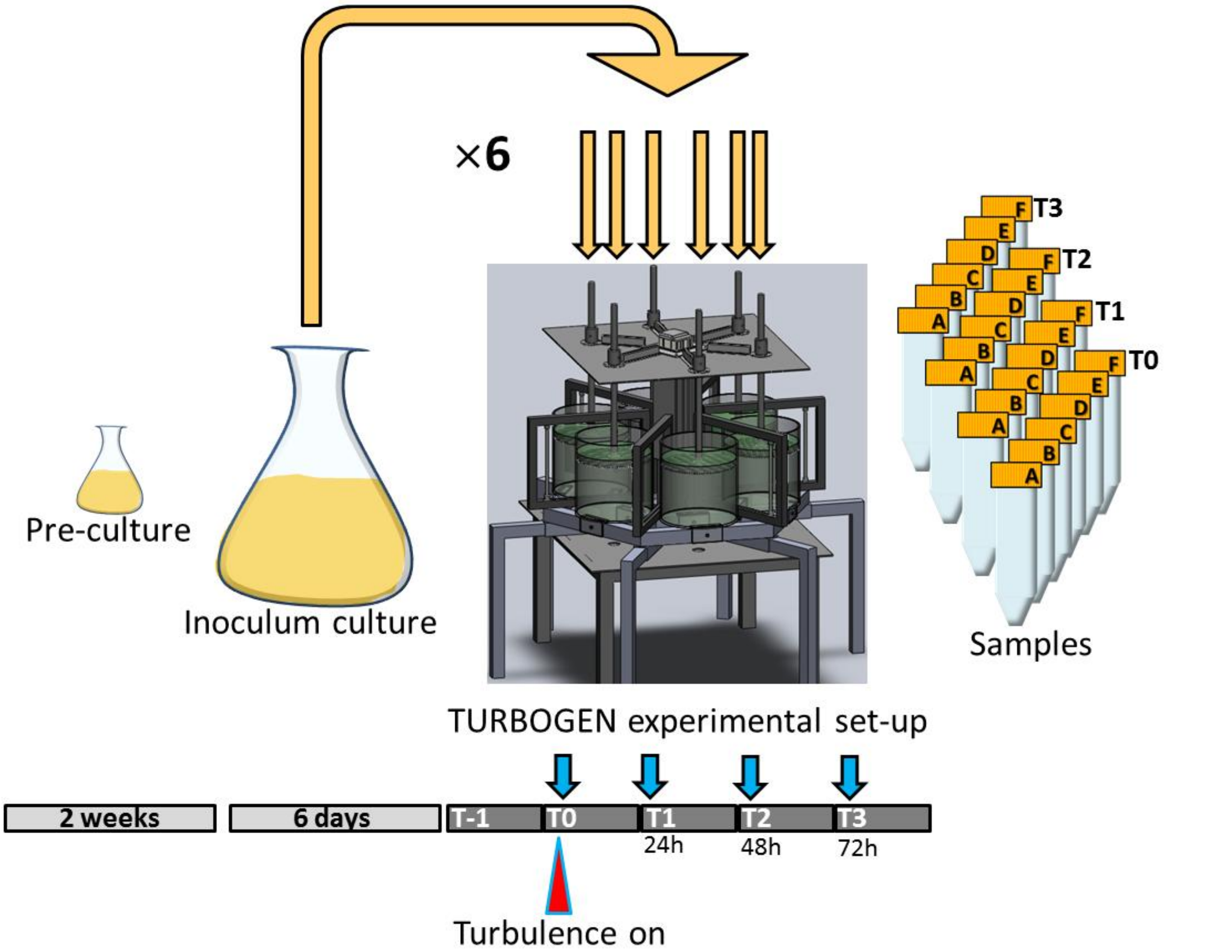

Supplementary Figure 1. Workflow of the experiments run in this investigation. Vertical golden arrows indicate diatom inoculum into the TURBOGEN[3]. Vertical blue arrows indicate sampling time points. The red arrowhead indicates the moment at which turbulence was applied in three out of the six cylinders. For more details about the experimental plan see Materials and Methods and Supplementary Methods.

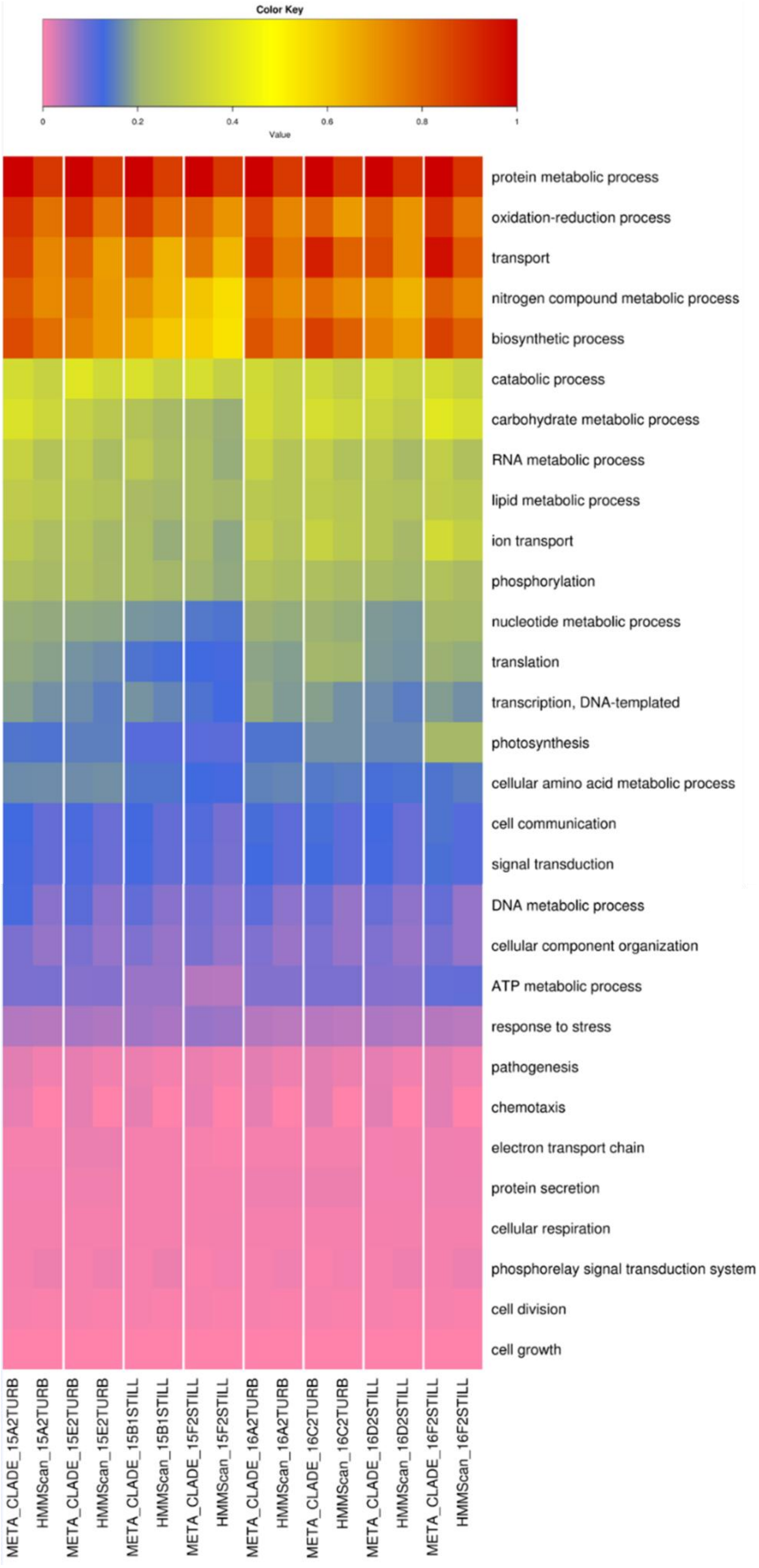

Supplementary Figure 2. Comparative table of domain abundance organized with respect to the domain classification based on GO-slim functional classes. The level of domain abundance (entries of the table) for each sample and each GO-slim functional class are described. Samples are arranged in eight columns reporting, for each functional class, the level of abundance obtained with META-CLADE and HMMScan. The color bar ranks high levels of abundance in dark red and very low levels in pink. The ranking of GO-slim functional classes (from top to bottom in the table) is fixed by the average abundance of domains in the eight samples detected by META-CLADE. Six GO-slim classes are not reported in the figure because they have no signal.

a

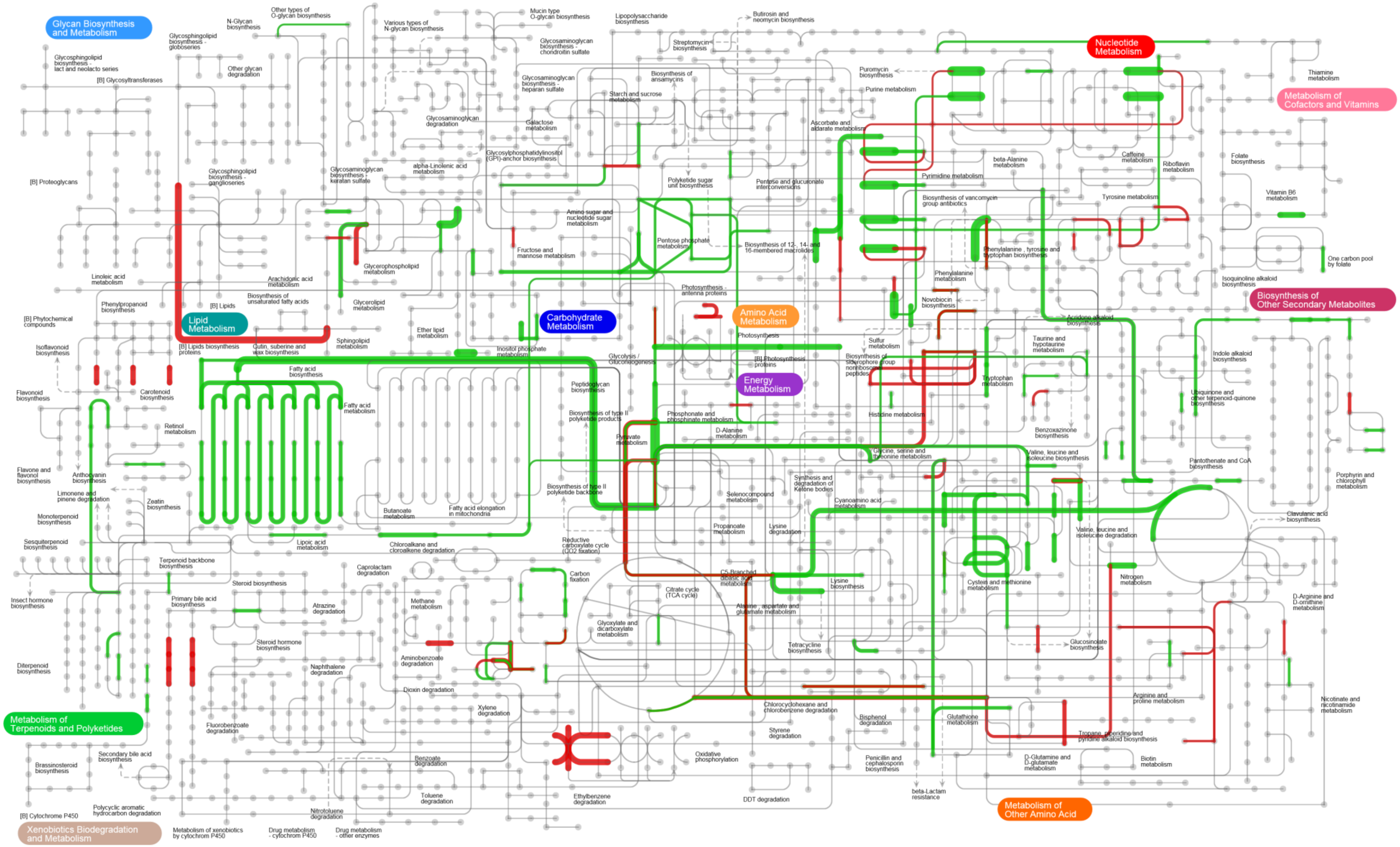

Glycan Biosynthesis and Metabolism
Nucleotide Metabolism
Metabolism of Cofactors and Vitamins
Biosynthesis of Other Secondary Metabolites
Lipid Metabolism
Carbohydrate Metabolism
Amino Acid Metabolism
Energy Metabolism
Metabolism of Terpenoids and Polyketides
Xenobiotics Biodegradation and Metabolism
Metabolism of Other Amino Acid
Purine metabolism
Pyrimidine metabolism
Thiamine metabolism
Riboflavin metabolism
Folate biosynthesis
Vitamin B6 metabolism
One carbon pool by folate
Pentose phosphate metabolism
Fructose and mannose metabolism
Galactose metabolism
Starch and sucrose metabolism
Glycolysis / Gluconeogenesis
Citrate cycle (TCA cycle)
Glyoxylate and dicarboxylate metabolism
Pyruvate metabolism
Propanoate metabolism
Butanoate metabolism
Fatty acid biosynthesis
Fatty acid metabolism
Fatty acid elongation in mitochondria
Glycerophospholipid metabolism
Glycerolipid metabolism
Sphingolipid metabolism
Arachidonic acid metabolism
Linoleic acid metabolism
alpha-Linolenic acid metabolism
Ether lipid metabolism
Steroid biosynthesis
Primary bile acid biosynthesis
Secondary bile acid biosynthesis
Steroid hormone biosynthesis
Oxidative phosphorylation
Photosynthesis
Photosynthesis - antenna proteins
Carbon fixation
Methane metabolism
Nitrogen metabolism
Sulfur metabolism
Tyrosine metabolism
Phenylalanine metabolism
Tryptophan metabolism
Histidine metabolism
Lysine biosynthesis
Lysine degradation
Arginine and proline metabolism
Glutathione metabolism
Taurine and hypotaurine metabolism
beta-Alanine metabolism
Cysteine and methionine metabolism
Glycine, serine and threonine metabolism
Valine, leucine and isoleucine biosynthesis
Valine, leucine and isoleucine degradation
Alanine, aspartate and glutamate metabolism
Porphyrin and chlorophyll metabolism
Pantothenate and CoA biosynthesis
Nicotinate and nicotinamide metabolism
Biotin metabolism
Carotenoid biosynthesis
Terpenoid backbone biosynthesis
Flavonoid biosynthesis
Phenylpropanoid biosynthesis
Benzoate degradation
Toluene degradation
Xylene degradation
DDT degradation
Metabolism of xenobiotics by cytochrome P450
Drug metabolism - cytochrom P450
Drug metabolism - other enzymes

b

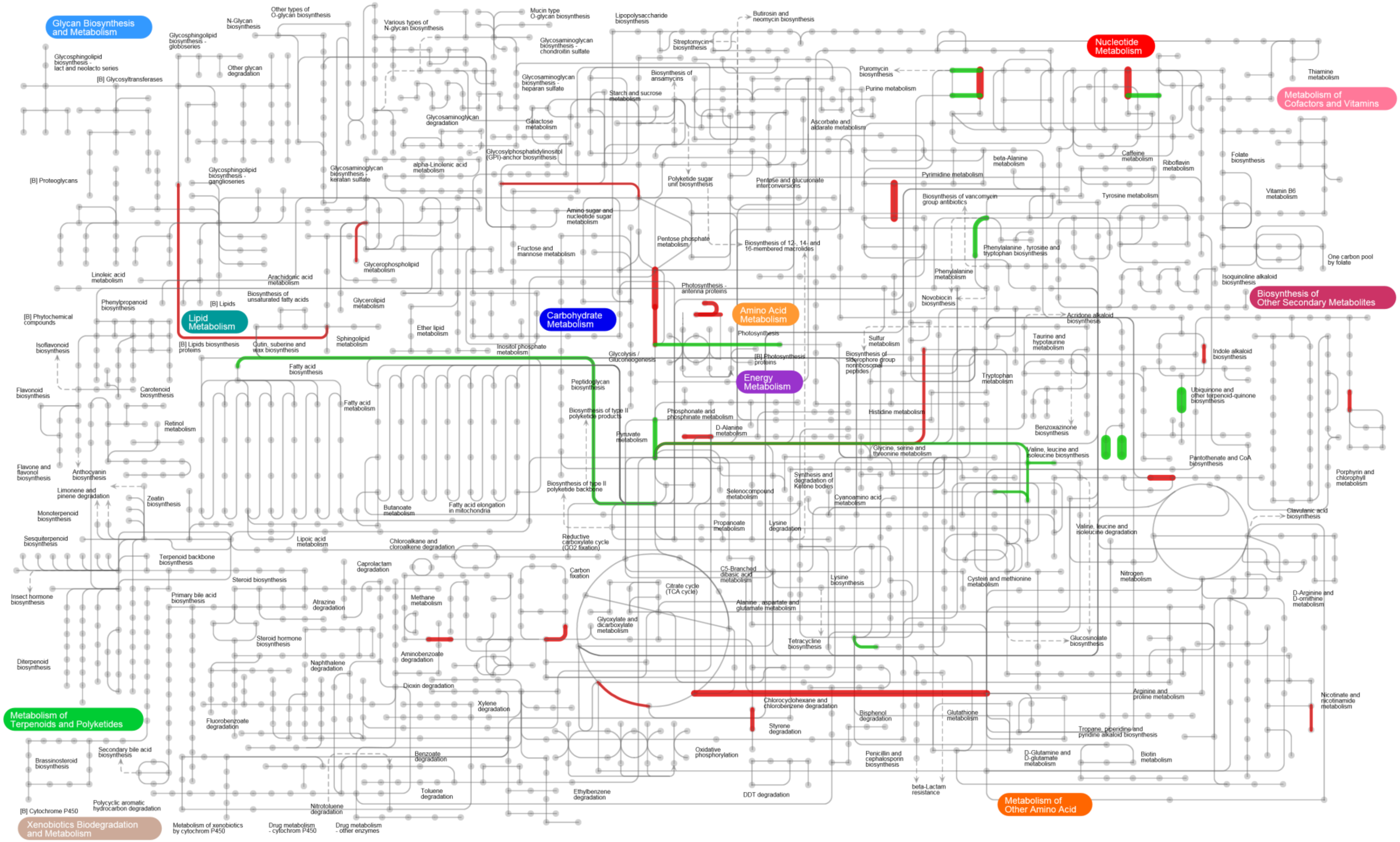

Glycan Biosynthesis and Metabolism
Nucleotide Metabolism
Metabolism of Cofactors and Vitamins
Lipid Metabolism
Carbohydrate Metabolism
Amino Acid Metabolism
Energy Metabolism
Biosynthesis of Other Secondary Metabolites
Metabolism of Terpenoids and Polyketides
Xenobiotics Biodegradation and Metabolism
Metabolism of Other Amino Acid
N-Glycan biosynthesis
Other types of O-glycan biosynthesis
Various types of N-glycan biosynthesis
Mucin type O-glycan biosynthesis
Lipopolysaccharide biosynthesis
Butirosin and neomycin biosynthesis
Glycosphingolipid biosynthesis - globoseries
Glycosphingolipid biosynthesis - lact and neolacto series
[B] Glycosyltransferases
Other glycan degradation
Glycosaminoglycan biosynthesis - chondroitin sulfate
Streptomycin biosynthesis
Glycosaminoglycan biosynthesis - heparan sulfate
Biosynthesis of ansamycins
Puromycin biosynthesis
Purine metabolism
Thiamine metabolism
Starch and sucrose metabolism
Glycosaminoglycan degradation
Galactose metabolism
Ascorbate and aldarate metabolism
[B] Proteoglycans
Glycosphingolipid biosynthesis - ganglioseries
Glycosaminoglycan biosynthesis - keratan sulfate
alpha-Linolenic acid metabolism
Glycosylphosphatidylinositol (GPI)-anchor biosynthesis
Polyketide sugar unit biosynthesis
Pentose and glucuronate interconversions
Pyrimidine metabolism
beta-Alanine metabolism
Caffeine metabolism
Riboflavin metabolism
Folate biosynthesis
Biosynthesis of vancomycin group antibiotics
Tyrosine metabolism
Vitamin B6 metabolism
Amino sugar and nucleotide sugar metabolism
Fructose and mannose metabolism
Pentose phosphate metabolism
Biosynthesis of 12-, 14- and 16-membered macrolides
Phenylalanine, tyrosine and tryptophan biosynthesis
One carbon pool by folate
Linoleic acid metabolism
Arachidonic acid metabolism
Glycerophospholipid metabolism
Phenylalanine metabolism
Isoquinoline alkaloid biosynthesis
[B] Lipids
Biosynthesis of unsaturated fatty acids
Glycerolipid metabolism
Photosynthesis - antenna proteins
Novobiocin biosynthesis
Phenylpropanoid biosynthesis
[B] Phytochemical compounds
Acridone alkaloid biosynthesis
Ether lipid metabolism
Photosynthesis
Sulfur metabolism
Taurine and hypotaurine metabolism
Isoflavonoid biosynthesis
[B] Lipids biosynthesis proteins
Cutin, suberine and wax biosynthesis
Sphingolipid metabolism
Inositol phosphate metabolism
Glycolysis / Gluconeogenesis
[B] Photosynthesis proteins
Biosynthesis of siderophore group nonribosomal peptides
Indole alkaloid biosynthesis
Fatty acid biosynthesis
Tryptophan metabolism
Flavonoid biosynthesis
Carotenoid biosynthesis
Peptidoglycan biosynthesis
Ubiquinone and other terpenoid-quinone biosynthesis
Fatty acid metabolism
Biosynthesis of type II polyketide products
Phosphonate and phosphinate metabolism
Histidine metabolism
Retinol metabolism
D-Alanine metabolism
Benzoxazinone biosynthesis
Pyruvate metabolism
Glycine, serine and threonine metabolism
Valine, leucine and isoleucine biosynthesis
Flavone and flavonol biosynthesis
Anthocyanin biosynthesis
Pantothenate and CoA biosynthesis
Porphyrin and chlorophyll metabolism
Synthesis and degradation of Ketone bodies
Limonene and pinene degradation
Biosynthesis of type II polyketide backbone
Selenocompound metabolism
Cyanoamino acid metabolism
Zeatin biosynthesis
Butanoate metabolism
Fatty acid elongation in mitochondria
Monoterpenoid biosynthesis
Clavulanic acid biosynthesis
Propanoate metabolism
Lysine degradation
Valine, leucine and isoleucine degradation
Sesquiterpenoid biosynthesis
Lipoic acid metabolism
Chloroalkane and chloroalkene degradation
Reductive carboxylate cycle (CO2 fixation)
Terpenoid backbone biosynthesis
Caprolactam degradation
Carbon fixation
C5-Branched dibasic acid metabolism
Lysine biosynthesis
Cysteine and methionine metabolism
Nitrogen metabolism
Steroid biosynthesis
Citrate cycle (TCA cycle)
Insect hormone biosynthesis
Primary bile acid biosynthesis
Atrazine degradation
Methane metabolism
Alanine, aspartate and glutamate metabolism
D-Arginine and D-ornithine metabolism
Glyoxylate and dicarboxylate metabolism
Steroid hormone biosynthesis
Tetracycline biosynthesis
Glucosinolate biosynthesis
Aminobenzoate degradation
Diterpenoid biosynthesis
Naphthalene degradation
Dioxin degradation
Xylene degradation
Chlorocyclohexane and chlorobenzene degradation
Arginine and proline metabolism
Nicotinate and nicotinamide metabolism
Fluorobenzoate degradation
Bisphenol degradation
Glutathione metabolism
Styrene degradation
Tropane, piperidine and pyridine alkaloid biosynthesis
Secondary bile acid biosynthesis
Brassinosteroid biosynthesis
Benzoate degradation
Oxidative phosphorylation
Penicillin and cephalosporin biosynthesis
D-Glutamine and D-glutamate metabolism
Biotin metabolism
Toluene degradation
Ethylbenzene degradation
DDT degradation
beta-Lactam resistance
[B] Cytochrome P450
Polycyclic aromatic hydrocarbon degradation
Nitrotoluene degradation
Metabolism of xenobiotics by cytochrome P450
Drug metabolism - cytochrome P450
Drug metabolism - other enzymes

Supplementary Figure 3. iPath representations of DE transcripts a) at time point T2 b) and time point T3. Green lines indicate up-regulation in turbulence-exposed cells compared to still cultures. Red lines indicate down-regulation. The thickness of the lines is proportional to the extent of the change.

Supplementary Tables

Legend to the Supplementary Table 1

Supplementary Table 1 contains the output of the differential expression analyses performed at time point T2. The table is arranged as follows: each row is a transcript and columns contain transcript features.

TranscriptName: is the name of the transcript as given in the FASTA file

logFC: Fold Change in log scale, a positive FC means higher expression in turbulence, a negative FC higher expression in still

PValue: P value for significance

FDR: False Discovery Rate

cpm_T2_B1_STILL, cpm_T2_F2_STILL, cpm_T2_A2_TURB, cpm_T2_E2_TURB: counts per million for the two still and two turbulent samples respectively

HSPNameSP: this is the first HSP result (lowest e-value) as given from the BLASTx output against Swiss-Prot

HSPEvalueSP: is the corresponding e-value assigned to the HSP

DescriptionSP: description of the HSP

EnzymeDescs: enzyme descriptions of the EnzymeIDs

Pathways: Pathways of first level associated to the transcripts as they are found in UniPathway

DescriptionUf: description of the HSP against UniRef

HSPEvalueUf: the corresponding e-value assigned to the HSP

Taxonomy: taxonomy corresponding to the result of UniRef

BPDesc: Biological processes descriptions

MFDesc: molecular descriptions

CCDesc: cellular components descriptions

AC_pfamID: pfam IDs

AC_GOid: Gene Ontology (GO) IDs

AC_GOddesc: GO descriptions

CDDesc: domains descriptions

Legend to the Supplementary Table 2

Supplementary Table 2 contains the output of the differential expression analyses performed at time point T3. The table is arranged as described for Supplementary Table 1.

Supplementary Table 3. **Selection of regulated transcripts at T2 and T3** grouped by the most represented KO pathways. Supplementary Tables 1 and 2 show the complete dataset. Differential expression (log fold change) and False Discovery Rate are indicated for each experimental point. Annotation and best hit of a tblastn search in NCBI are reported. Green shades indicate up-regulation, red-shades indicate down-regulation. Darker nuances indicate up-regulation above 1.5 or down-regulation below -1.5 (arbitrarily set cut-off values). For all the transcripts reported here a tblastn search in MMETSP database was performed and the first 50 hits are listed in Supplementary Table 11.

**Level 2 KO pathways**	**Transcript**	**Annotation and best hit**	**T2**		**T3**	
			logFC	FDR	logFC	FDR
Calvin cycle	c8596	SBPase XM_002176982.1	1.30	1.76E-03		
	c10813	PRK Y08610.2	1.30	8.77E-04		
	c14492	chl-PGK XM_002288305.1	1.24	3.95E-04		
	c6859	PGK precursor AF108451.1	-1.31	3.27E-04	-1.08	2.12E-02
fatty acid biosynthesis	c4257	FabF XM_002181417.1	2.23	4.81E-07		
	c9683	FabI XM_002295703.1	2.15	4.55E-07		
	c6844	(3R)-3-hydroxyacyl-CoA dehydratase XM_002295703.1	2.15	2.95E-07		
	c14925	FAS2/FabB AY099587.1	1.98	2.75E-07		
	c5520	FabG XM_002180866.1	1.83	3.94E-06		
	c5253	FabH XM_002295284.1	1.47	3.37E-05		
	c1415	FabD XM_002181731.1	1.41	3.38E-04		
	c3263	Enoyl-ACP reductase AY826870.1	1.28	1.69E-03		

	c1981	PTER XM_005855553.1	1.05	2.72E-02		
polyunsaturated fatty acid biosynthesis	c9333	Elongase XM_002288445.1	1.56	6.89E-05		
	c9138	Omega-6 fatty acid desaturase XM_001569292.1	1.48	3.80E-05		
	c10316	Omega-6 fatty acid desaturase AY165024.1	1.38	2.35E-04		
	c9857	Delta(12) fatty acid desaturase AB858398.1	1.05	6.98E-03		
malonyl-CoA biosynthesis	c10543	Acetyl-CoA carboxylase KF673101.1	3.84	2.12E-14	1.53	1.34E-02
	c10935	Acetyl-CoA carboxylase XM_002296047.1	1.96	4.90E-06		
glycolysis	c13267	Pyruvate kinase XM_002289369.1	4.37	1.45E-10	2.19	2.09E-03
	c7832	Pyruvate kinase XM_002289831.1	3.47	1.02E-12	1.87	5.82E-04
	c7622	Enolase XM_002295703.1	2.62	1.78E-08		
	c10371	GPI 3 XM_002185239.1	2.17	9.73E-06		
	c7193	Fba IXM_005840665.1	1.75	2.57E-06		
	c10844	Fba C1 XM_002176146.1	1.68	5.68E-05		
	c5521	ENO1 XM_002289830.1	1.63	9.76E-06		
	c5369	PGAM6 XM_002178483.1	1.48	4.48E-05		
	c5850	Fba C2 XM_002183735.1	1.18	4.54E-03		
	c10803	PK1 XM_002182782.1	1.09	4.30E-03		

	c16505	Phosphoglycerate kinase XM_004532595.1	-1.20	3.09E-02		
	c9688	GAPDH AF063801.1	-2.13	4.00E-03		
	c4834	GapC3 precursor AF063802.1	1.18	8.03E-03	1.28	1.90E-02
	c17273*	GAPDH CP004349.1			-3.72	7.85E-04
pentose phosphate pathway	c8818	6-phosphogluconolactonase XM_002182523.1	2.20	3.35E-07		
	c3274	TAL XM_002185209.1	1.34	1.33E-03		
	c10651	Transketolase XM_002287164.1	1.25	7.13E-03		
L-tryptophan biosynthesis	c16748	TrpAB XM_002294670.1	1.08	3.89E-03		
	c3306	TRP1 AY030294.1	1.44	1.31E-03		
isopentenyl diphosphate biosynthesis via DXP pathway	c7546	CMK XM_002287613.1	2.26	2.23E-06		
	c13504	DXR XM_002176918.1	1.72	9.34E-06		
	c7234	HDS FJ175678.1	1.48	3.17E-05		
	c2948	XM_005820425.1	1.35	3.02E-04		
farnesyl diphosphate biosynthesis geranyl diphosphate biosynthesis	c10583	KM360174.1	1.44	2.87E-04		
	c7924	XM_002288303.1	1.35	1.59E-		

				04		

* tblastn hits are all from bacteria

Supplementary Table 4. Primers for qPCR validations. Transcript ID, primer sequence, primer length (bp) and amplicon length are reported.

**Transcript**	**Forward primer**	**bp**	**Reverse primer**	**bp**	**Amplicon length (bp)**
c2730	CCATCTCAGCACTTGGAACA	20	ACATGACGAGCAAGCATGAG	20	199
c13267	AAACAAGTCACCCTCGTTGG	20	CCAAATGTTCCAATCGCAGT	20	177
c2115	AGGAAGCACAAGCAAAGCTC	20	CAAGGACTTCCCATCCAATC	20	189
c14633	CCTGGTGCAGCTCGGATA	18	TGATTCAAGAAGGTCAGCAATG	22	105
c71	GTGAGTTTGCCATCCTTGTC	20	CAATGGAATGGTTTCTGGTT	20	168
c16363	GCAATTTCCTTCTCTGCATT	20	TGCTTTCTGGACCTGTTGTT	20	190
c2210	AAAGCAGGACATGAACCAGA	20	GCGCTATACCCATCTTTCCT	20	169
c17476	CGCCTTAATCCAACACAAAC	20	TATTTCCACCCAAACGAACA	20	202
c5660	TAGGAATCCGAGGCACTACA	20	GCACAAGTCATGGCAACTC	19	175
c7202	AATCCTGAAACCCAGAGTGA	20	GAGCCTATCCATTCCTCCA	19	207
c17770	ATCAGTTGCTTGCTGTTGGT	20	CCTCAGATGGAGAAGGTGTC	20	248
c5179	GATGTTCCAGGGTGTTCTTG	20	AACTGCTTCAAATCCGCTAC	20	219
c991	ACCACTTCATGTTGCTGCAC	20	AGCGGAAGACATGAAGAAGC	20	207
c11391	CCAAGTCCATCCTCTTTGGA	20	AGCGGAAGACATGAAGAAGC	20	181
c17437	ACGTAGTGGTGATGCCAAACT	21	CATCTTACCCGCAGCTCTCT	20	193
c12330	CCGTAACATTTGCGTTAGAAG	21	GCGTCATCAAGTGTGGTAAA	20	235
c10008 (CDK-A)	GAATGGCTGTACTTGGGATG	20	CAACAATGGAACGGTATCAAA	21	153
c6390 (TBP)	TGCACTCAAATGCCGAAATA	20	TCACTGGTTGTGTCTTGAATCC	22	206
c5402 (TUBb)	AGTTCAGCTCCCTCGGTGTA	20	TTCCAAGGGCAATCCTAATG	20	157

Supplementary Table 5. qPCR validation of a selection of transcripts either up or down regulated at time points T2 and T3. For qPCR RNA samples from all the cylinders were reverse transcribed and amplified with primer pairs listed in Supplementary Table 4. Transcript ID, Time point, Gene function (where available), qPCR and RNA-seq differential expression values (expressed as log2 fold change) are reported. For qPCR the differential expression is reported both as mean over the three cylinders and by cylinder.

Transcript ID	Time point	Gene Function	Log2 average relative expression	
			**qPCR (±SD)**	**RNA-seq**
C2730	T2	Uncharacterized protein	3.85 (0.64)<br>A = 4.37<br>C = 4.05<br>E = 3.13	4.37
C13267	T2	Pyruvate kinase	3.31 (0.57)<br>A = 3.72<br>C = 3.54<br>E = 2.66	4.41
C2115	T2	Predicted protein ion binding	3.16 (0.84)<br>A = 4.13<br>C = 2.72<br>E = 2.64	4.43
C14633	T2	-	3.07 (1.53)<br>A = 4.09<br>C = 3.81<br>E = 1.31	5.91
C71	T2	Tricarboxylate transport protein mitochondrial	3.53 (0.70)<br>A = 3.97<br>C = 3.90<br>E = 2.72	4.12
C16363	T2	Nucleoside diphosphate kinase	3.03 (0.95)<br>A = 3.46<br>C = 3.68<br>E = 1.94	4.10
C2210	T2	Predicted protein GTP binding protein	3.22 (0.80)<br>A = 3.86<br>C = 3.47<br>E = 2.33	4.08
C17476	T2	Hairpin binding protein 1	3.55 (0.66)<br>A = 3.99<br>C = 3.88	3.36

			E = 2.79	
C5660	T3	Uncharacterized protein triglyceride lipase activity	3.88 (1.05)<br>A = 2.96<br>C = 3.66<br>E = 5.03	4.97
C7202	T2	Predicted protein Glucosylceramidase	-2.90 (0.28)<br>A = -3.22<br>C = -2.75<br>E = -2.72	-3.18
C19910	T2	Uncharacterized protein transmembrane transporter	-3.97 (0.40)<br>A = -4.30<br>C = -3.53<br>E = -4.08	-4.25
C17770	T2	Uncharacterized protein	-3.79 (1.49)<br>A = -4.01<br>C = -2.20<br>E = -5.15	-3.00
C17770	T3	Uncharacterized protein	-6.04 (3.27)<br>A = -5.94<br>C = -9.36<br>E = -2.83	-7.44
C5179	T3	Frustulin 5	-4.71 (1.31)<br>A = -4.65<br>C = -6.05<br>E = -3.42	-3.76
c991	T3	Uncharacterized protein	1.17 (1.23)<br>A = 0.33<br>C = 0.61<br>E = 2.59	2.94
c11391	T3	Uncharacterised protein	0.35 (1.01)<br>A = -0.50<br>C = 0.07<br>E = 1.47	3.00
c17437	T3	Predicted protein	0.78 (0.93)<br>A = 0.07<br>C = 0.43<br>E = 1.84	3.69

c12330	T3	-	-0.47<br>(1.65)<br>A = -0.88<br>C = -1.88<br>E = 1.34	2.84

Supplementary Table 6. List of transcripts involved in isoprenoid biosynthesis, fatty acid β-oxidation and pentose phosphate and glycolysis pathways. Desaturases and elongase as reported by Dolch and Maréchal[18] were also added. Transcripts involved in fatty acid biosynthesis are listed in Supplementary Table 3. Green shades indicate up-regulation, red-shades indicate down-regulation. Darker nuances indicate up-regulation above 1.5 or down-regulation below -1.5 (arbitrary cut-off values). For all the transcripts reported here, except those marked with the symbol $^+$, a tBLASTn search in MMETSP database was performed and the first 50 hits are listed in Supplementary Table 11.

**Transcript**	**Annotation**	**Best hit**	**logFC**	**pfam**
Inositol pathway and signal transduction				
c19854$^+$	Inositol monophosphatase 3	XM_002502522.1	4.2	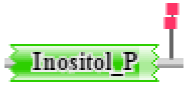
c12037$^+$	Phosphatidylinositol 4-kinase	XM_002286092.1	1.9	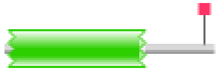 PI3_PI4_kinase
c13992$^+$	Phosphatidylinositol 4-kinase	XM_013307010.1	1.8	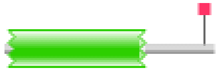 PI3_PI4_kinase
c16363$^+$	NDP kinase		4.06	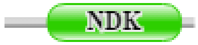
c1791$^+$	NDP kinase		1.70	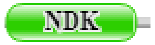
c787$^+$	NDP kinase		1.30	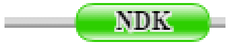
MVA pathway (isoprenoid biosynthesis)				
c2017	HMGS	XM_002286531.1	-	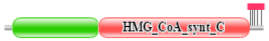
c8934	HMGR	XM_004337355.1	-	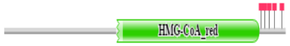

c12	MPDC	XM_002286995.1	-	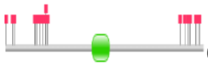 GHMP kinases N terminal domain
c7602	DXS	XM_002176350.1	1.01	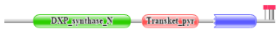
MPE pathway (isoprenoid biosynthesis)				
c13504	DXR (DXR domains)	XM_002176918.1	1.72	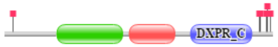
c7546	CMK	XM_002287613.1	2.26	no domains in Tp
c1881	MDS	XM_005826781.1	-	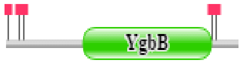
c7234	HDS (GcpE protein)	FJ175678.1	1.48	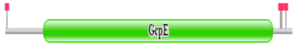
c7419	HDR	XM_002178581.1	-	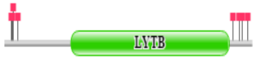
c2948	2-C-methyl-D-erythritol 4-phosphate cytidylyltransferase	XM_005820425.1	1.35	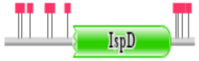
c10583	Polyprenyl synthetase	KM360174.1 XM_002185991.1	1.44	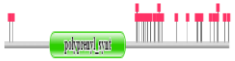
c7924	Polyprenyl synthetase	XM_002288303.1	1.35	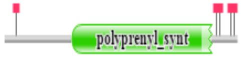
Fatty acids β-oxidation				

c3617	KCT3<br>Enoyl-CoA hydratase/isomerase<br>3-hydroxyacyl-CoA dehydrogenase, NAD binding domain<br>3-hydroxyacyl-CoA dehydrogenase, C-terminal domain)	Phatr_35240[18]	-	
c1094	acyl-CoA dehydrogenase	Phatr_11014[18]	-	
c7189	enoyl-CoA hydratase	Phatr_55192[18]	-	
c3358	3-ketoacyl-CoA dehydrogenase	Phatr_54947[18]	-	
Desaturase				
c9857	FAD2	Phatr_25769[1]	1.05	
c10316	FAD6	Phatr_48423[1]	1.4	
c10828	ERΔ5FAD.1/PTD5b	Phatr_46830[1]	1.4	
c8233	PTD6	XM_002291493.1	1.30	
c9138	PlastidΔ6FAD	Phatr_50443[1]	1.48	
c8389	PAD/SAD	Phatraft_9316[18]	-	
c10330	ADS	Phatr_28797[18]	-	
c10282	ERΔ5FAD.2*	Phatr_22459[18]	-	

c862	ERΔ4FAD	Phatr_22510[18]	-	
c7981	Plastidω3FAD/FAD7/PTD 15	Phatr_41570[18]	-	
c5093	FAD4	Phatr_41301[18]	-	
c9333	Elongase	XM_002288445.1	1.56	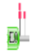
Pentose Phosphate and Glycolysis Pathways				
c10371	Glucose-6-phosphate isomerase	XM_002185239.1	2.13	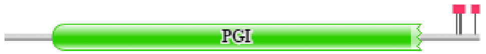
c10844	Fructose-1,6 biphosphate aldolase	XM_002176146.1	1.68	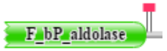
c5850	Fructose-1,6 biphosphate aldolase	XM_002288284.1	1.1	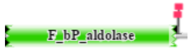
c3274	Transaldolase	XM_002185209.1	1.33	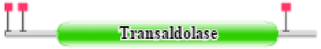
c7602	1-deoxy-D-xylulose-5-phosphate synthase	XM_002176350.1	1.00	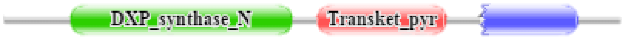
c10651	Transketolase	XM_002287164.1	1.00	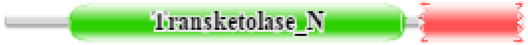
c5034	triosephosphate isomerase	XM_002178067.1	1.4	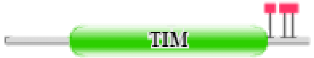
c9233	triosephosphate isomerase	XM_002177666.1	1.6	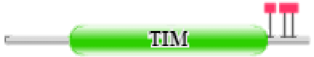

c4834	Glyceraldehyde-3-phosphate dehydrogenase	AF063802.1	1.18	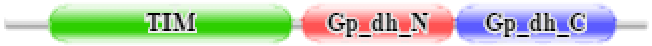
c8818	6-phosphogluconolactonase	XM_002182523.1	2.19	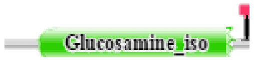
c9012^{+}	6-phosphogluconate dehydrogenase	XM_002289784.1	-	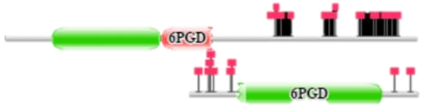
c10101^{+}	fructose 6-phosphate 1-phosphotransferase	AF268276.1	-	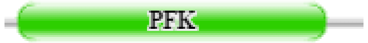
c18606^{+}	Ribose-phosphate pyrophosphokinase	XM_002287674.1	-	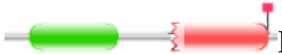N-terminal domain of ribose phosphate pyrophosphokinase and Phosphoribosyl synthetase-associated domain
c5366^{+}	Ribose-phosphate pyrophosphokinase	XM_002180827.1	-	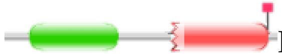N-terminal domain of ribose phosphate pyrophosphokinase and Phosphoribosyl synthetase-associated domain
c4769^{+}	ribose-5-phosphate isomerase	XM_002287269.1	-	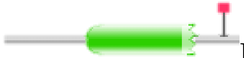Ribose 5-phosphate isomerase
c6782^{+}	ribulose-phosphate 3-epimerase	XM_002179628.1	-	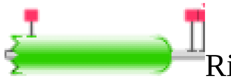Ribulose-phosphate 3 epimerase
c19160^{+}	Fructose-1 6-bisphosphatase	XM_007512729.1	-	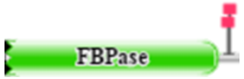

c9258[+]	FGGY carbohydrate kinase domain-containing protein	XM_003057750.1	-	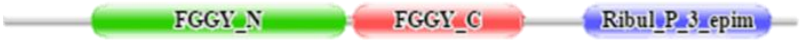
c10668[+]	Fructose-1 6-bisphosphatase	XM_002176491.1	-	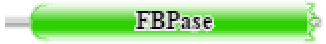
c19105[+]	Fructose-1 6-bisphosphatase	XM_002176491.1	-	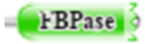

* ERΔ5FAD.1 and ERΔ5FAD.2 have a high level of identity between each other and thus it is difficult to tell which is which. ERΔ5FAD.1 was functionally validated[18]

[+] Not blasted in the MMETSP database

Supplementary Table 7. Cell concentration expressed as cells·ml^{-1} estimated by Sedgewick-Rafter counts (Supplementary Methods) for *Chaetoceros decipiens* Experiment 1 and Experiment 2, *Thalassiosira rotula* and *Skeletonema marinoi*. At the moment of inoculum (T-1), cell concentrations were set at 250, 500, and 2.5×10^{3} cell·ml^{-1}, respectively (see Materials and Methods). Average (Avg) and standard deviations (σ) are reported. Cell concentration data were used to build growth curves (Fig. 2a-d) and log$_{2}$-transformed to calculate division rates.

**Time point**	***C. decipiens* experiment 1**									
	**Turbulent**					**Still**				
	**A**	**C**	**E**	**Avg**	**σ**	**B**	**D**	**F**	**Avg**	**σ**
**T0**	469	609	518	532	71.3	507	512	429	483	46.4
**T1**	518	583	594	565	41.3	730	1215	977	974	242.7
**T2**	1157	1218	1628	1334	256.0	2430	1908	2217	2185	262.5
**Time point**	***C. decipiens* experiment 2**									
	**Turbulent**					**Still**				
	**A**	**C**	**E**	**Avg**	**σ**	**B**	**D**	**F**	**Avg**	**σ**
**T0**	495	492	472	486	12.8	518	795	544	619	153.0
**T1**	600	676	408	561	138.4	841	538	676	685	152.0
**T2**	1033	1059	917	1003	75.9	1725	1158	1672	1518	313.4
**T3**	1478	1818	1445	1581	206.3	3053	2538	2226	2606	417.6
**Time point**	***Thalassiosira rotula***									
	**Turbulent**					**Still**				
	**A**	**C**	**E**	**Avg**	**σ**	**B**	**D**	**F**	**Avg**	**σ**
**T0**	718	546	743	669	107.2	670	599	577	615	48.8
**T1**	883	1171	810	954	191.0	1987	1521	1508	1672	272.8
**T2**	3134	2245	1784	2388	686.5	5829	3722	3149	4233	1411.2
**T3**	4215	3471	3284	3657	492.6	8580	5225	5868	6558	1780.8
**Time point**	***Skeletonema marinoi***									
	**Turbulent**					**Still**				
	**A**	**C**	**E**	**Avg**	**σ**	**B**	**D**	**F**	**Avg**	**σ**
**T0**	5350	5500	5050	5300	229.1	5150	5100	5100	5117	28.9
**T1**	22000	19000	20000	20333	1527.5	21000	18000	18500	19167	1607.3
**T2**	24900	26700	31200	27600	3245.0	27900	26600	27200	27233	650.6
**T3**	32700	31500	28100	30767	2386.0	28100	34200	43100	35133	7543.4

Supplementary Table 8. Non-parametric statistic tests (Kolmogorov-Smirnov and Wilcoxon). The letters indicate TURBOGEN cylinders: A, C, and E turbulent conditions; B, D, and F still conditions. First six columns (Controls, in red) show internal controls, i.e. turbulent vs turbulent replicates (A-C, A-E, and C-E) and still vs still replicates (B-D, B-F, and D-F). In red bold-face underlined digits, the internal controls that reject null hypothesis. TX (0≥X≥3) indicates time points. ‘h’ indicates the test answer: 0 = null hypothesis accepted; 1 = null hypothesis consistently rejected. ‘p’ indicates the p-value associated to the test outcome. Accuracy set at 99.9% ($\alpha = 10^{-3}$).

Chaetoceros decipiens Experiment 1 **Kolmogorov-Smirnov**															
	**Controls**						**Turbulent vs still**								
**T0**	**AC**	**AE**	**CE**	**BD**	**BF**	**DF**	**AB**	**AD**	**AF**	**CB**	**CD**	**CF**	**EB**	**ED**	**EF**
h	0	0	0	0	0	0	0	0	0	0	0	0	0	0	0
p	1	0.74	0.89	0.93	0.70	0.47	0.98	1	0.93	1	0.97	0.97	0.5598	0.2805	0.9762
**T1**	**AC**	**AE**	**CE**	**BD**	**BF**	**DF**	**AB**	**AD**	**AF**	**CB**	**CD**	**CF**	**EB**	**ED**	**EF**
h	0	0	0	0	0	0	0	0	0	0	0	1	0	0	0
p	0.25	0.57	0.20	0.41	0.37	0.37	0.21	0.21	$1.3 \cdot 10^{-3}$	0.02	0.09	$2.47 \cdot 10^{-5}$	0.83	0.08	0.02
**T2**	**AC**	**AE**	**CE**	**BD**	**BF**	**DF**	**AB**	**AD**	**AF**	**CB**	**CD**	**CF**	**EB**	**ED**	**EF**
h	0	0	0	0	0	0	1	1	1	1	1	1	1	1	1
p	0.04	0.32	0.02	0.60	0.12	0.75	$4.16 \cdot 10^{-5}$	$4.91 \cdot 10^{-6}$	$6.16 \cdot 10^{-4}$	$1.67 \cdot 10^{-9}$	$6.81 \cdot 10^{-9}$	$2.26 \cdot 10^{-6}$	$8.26 \cdot 10^{-4}$	$6.39 \cdot 10^{-6}$	$6.87 \cdot 10^{-4}$
Chaetoceros decipiens Experiment 1 **Wilcoxon**															
**T0**	**AC**	**AE**	**CE**	**BD**	**BF**	**DF**	**AB**	**AD**	**AF**	**CB**	**CD**	**CF**	**EB**	**ED**	**EF**
h	0	0	0	0	0	0	0	0	0	0	0	0	0	0	0
p	0.91	0.12	0.12	0.53	0.19	0.56	0.75	0.78	0.38	0.63	0.85	0.41	0.04	0.22	0.45
**T1**	**AC**	**AE**	**CE**	**BD**	**BF**	**DF**	**AB**	**AD**	**AF**	**CB**	**CD**	**CF**	**EB**	**ED**	**EF**
h	0	0	0	0	0	0	0	0	0	0	0	1	0	0	0
p	0.21	0.29	0.01	0.33	0.13	0.02	0.12	0.70	$3.4 \cdot 10^{-3}$	$5.9 \cdot 10^{-3}$	0.15	$5.13 \cdot 10^{-5}$	0.60	0.50	0.04
**T2**	**AC**	**AE**	**CE**	**BD**	**BF**	**DF**	**AB**	**AD**	**AF**	**CB**	**CD**	**CF**	**EB**	**ED**	**EF**
h	0	0	**<u>1</u>**	0	0	0	1	1	1	1	1	1	1	1	0
p	4.8	0.36	**<u>3.54</u>**	0.59	0.09	0.28	1.10	7.76	6.05	1.04	5.77	1.31	2.27	3.62	0.01

	$\cdot 10^{-3}$		**$\cdot 10^{-4}$**				$\cdot 10^{-7}$	$\cdot 10^{-6}$	$\cdot 10^{-4}$	$\cdot 10^{-13}$	$\cdot 10^{-11}$	$\cdot 10^{-8}$	$\cdot 10^{-5}$	$\cdot 10^{-4}$	
Chaetoceros decipiens Experiment 2 **Kolmogorov-Smirnov**															
**T0**	**AC**	**AE**	**CE**	**BD**	**BF**	**DF**	**AB**	**AD**	**AF**	**CB**	**CD**	**CF**	**EB**	**ED**	**EF**
h	0	0	0	0	0	0	0	0	0	0	0	0	0	0	0
p	0.99	0.98	0.47	1	0.45	0.73	0.76	0.98	0.72	0.91	1	0.99	0.14	0.32	0.12
**T1**	**AC**	**AE**	**CE**	**BD**	**BF**	**DF**	**AB**	**AD**	**AF**	**CB**	**CD**	**CF**	**EB**	**ED**	**EF**
h	0	0	0	0	0	0	1	0	0	0	0	0	0	0	0
p	0.39	0.03	0.91	0.12	0.95	0.44	2.41 $\cdot 10^{-4}$	0.30	3.7 $\cdot 10^{-3}$	0.10	0.99	0.42	0.45	0.90	0.78
**T2**	**AC**	**AE**	**CE**	**BD**	**BF**	**DF**	**AB**	**AD**	**AF**	**CB**	**CD**	**CF**	**EB**	**ED**	**EF**
h	0	**1**	0	0	0	0	1	1	1	1	1	1	1	0	0
p	4.2 $\cdot 10^{-3}$	**4.30 $\cdot 10^{-6}$**	0.05	0.33	0.52	1	6.37 $\cdot 10^{-21}$	9.31 $\cdot 10^{-18}$	1.24 $\cdot 10^{-15}$	5.49 $\cdot 10^{-9}$	9.19 $\cdot 10^{-7}$	1.28 $\cdot 10^{-5}$	1.80 $\cdot 10^{-4}$	3.7 $\cdot 10^{-3}$	0.02
**T3**	**AC**	**AE**	**CE**	**BD**	**BF**	**DF**	**AB**	**AD**	**AF**	**CB**	**CD**	**CF**	**EB**	**ED**	**EF**
h	**1**	0	0	0	0	0	1	1	1	1	1	1	1	1	1
p	**1.59 $\cdot 10^{-6}$**	0.01	0.28	0.89	0.98	1	2.48 $\cdot 10^{-40}$	3.75 $\cdot 10^{-31}$	2.79 $\cdot 10^{-34}$	2.46 $\cdot 10^{-18}$	3.49 $\cdot 10^{-19}$	6.68 $\cdot 10^{-20}$	6.40 $\cdot 10^{-22}$	2.38 $\cdot 10^{-22}$	2.71 $\cdot 10^{-23}$
Chaetoceros decipiens Experiment 2 **Wilcoxon**															
**T0**	**AC**	**AE**	**CE**	**BD**	**BF**	**DF**	**AB**	**AD**	**AF**	**CB**	**CD**	**CF**	**EB**	**ED**	**EF**
h	0	0	0	0	0	0	0	0	0	0	0	0	0	0	0
p	0.45	0.86	0.35	0.92	0.22	0.18	0.74	0.81	0.11	0.69	0.61	0.39	0.64	0.69	0.07
**T1**	**AC**	**AE**	**CE**	**BD**	**BF**	**DF**	**AB**	**AD**	**AF**	**CB**	**CD**	**CF**	**EB**	**ED**	**EF**
h	0	0	0	0	0	0	1	0	1	0	0	0	0	0	0
p	0.04	0.01	0.68	0.09	0.47	0.31	3.30 $\cdot 10^{-6}$	0.01	1.34 $\cdot 10^{-4}$	0.01	0.46	0.07	0.03	0.74	0.16
**T2**	**AC**	**AE**	**CE**	**BD**	**BF**	**DF**	**AB**	**AD**	**AF**	**CB**	**CD**	**CF**	**EB**	**ED**	**EF**
h	**1**	**1**	0	0	0	0	1	1	1	1	1	1	1	0	0
p	**8.67**	**2.43**	0.01	0.06	0.111	0.83	6.66	1.13	1.69	6.24	5.31	4.81	2.28	0.02	1.25

	**$\cdot 10^{-4}$**	**$\cdot 10^{-8}$**					$\cdot 10^{-23}$	$\cdot 10^{-15}$	$\cdot 10^{-15}$	$\cdot 10^{-12}$	$\cdot 10^{-7}$	$\cdot 10^{-7}$	$\cdot 10^{-5}$		$\cdot 10^{-3}$
**T3**	**AC**	**AE**	**CE**	**BD**	**BF**	**DF**	**AB**	**AD**	**AF**	**CB**	**CD**	**CF**	**EB**	**ED**	**EF**
h	**1**	0	0	0	0	0	1	1	1	1	1	1	1	1	1
p	**$9.32 \cdot 10^{-6}$**	0.01	0.06	0.63	0.78	0.83	$1.63 \cdot 10^{-43}$	$1.35 \cdot 10^{-34}$	$2.02 \cdot 10^{-38}$	$1.38 \cdot 10^{-23}$	$9.82 \cdot 10^{-19}$	$1.09 \cdot 10^{-20}$	$3.54 \cdot 10^{-30}$	$5.69 \cdot 10^{-24}$	$1.83 \cdot 10^{-26}$
Thalassiosira rotula **Kolmogorov-Smirnov**															
**T0**	**AC**	**AE**	**CE**	**BD**	**BF**	**DF**	**AB**	**AD**	**AF**	**CB**	**CD**	**CF**	**EB**	**ED**	**EF**
h	0	0	0	0	0	0	0	0	0	0	0	0	0	0	0
p	0.69	1	0.91	0.63	0.86	0.09	1	0.49	0.41	0.44	0.02	0.97	1	0.27	0.97
**T1**	**AC**	**AE**	**CE**	**BD**	**BF**	**DF**	**AB**	**AD**	**AF**	**CB**	**CD**	**CF**	**EB**	**ED**	**EF**
h	0	0	0	0	**1**	**1**	0	0	1	0	0	1	0	0	1
p	0.10	0.03	0.99	0.38	**$3.75 \cdot 10^{-4}$**	**$7.04 \cdot 10^{-5}$**	0.51	0.01	$8.14 \cdot 10^{-6}$	0.23	1	$2.00 \cdot 10^{-5}$	0.15	0.91	$3.11 \cdot 10^{-7}$
**T2**	**AC**	**AE**	**CE**	**BD**	**BF**	**DF**	**AB**	**AD**	**AF**	**CB**	**CD**	**CF**	**EB**	**ED**	**EF**
h	0	0	**1**	0	0	0	0	1	0	1	1	0	0	0	0
p	$5.57 \cdot 10^{-5}$	0.02	**$7.62 \cdot 10^{-9}$**	0.14	$2.7 \cdot 10^{-3}$	0.01	0.04	$7.23 \cdot 10^{-4}$	0.94	$8.34 \cdot 10^{-10}$	$1.24 \cdot 10^{-9}$	$1.3 \cdot 10^{-3}$	0.93	0.83	0.01
**T3**	**AC**	**AE**	**CE**	**BD**	**BF**	**DF**	**AB**	**AD**	**AF**	**CB**	**CD**	**CF**	**EB**	**ED**	**EF**
h	**1**	0	0	0	**1**	0	1	1	1	0	1	1	1	1	1
p	**$6.85 \cdot 10^{-4}$**	0.50	0.01	$1.0 \cdot 10^{-3}$	**$6.47 \cdot 10^{-6}$**	0.71	$3.46 \cdot 10^{-9}$	$1.48 \cdot 10^{-22}$	$1.58 \cdot 10^{-26}$	0.04	$1.50 \cdot 10^{-8}$	$1.52 \cdot 10^{-10}$	$2.13 \cdot 10^{-6}$	$2.07 \cdot 10^{-8}$	$2.14 \cdot 10^{-20}$
Thalassiosira rotula **Wilcoxon**															
**T0**	**AC**	**AE**	**CE**	**BD**	**BF**	**DF**	**AB**	**AD**	**AF**	**CB**	**CD**	**CF**	**EB**	**ED**	**EF**
h	0	0	0	0	0	1	0	0	0	0	0	0	0	0	0
p	0.25	0.69	0.65	0.06	0.08	$4.84 \cdot 10^{-4}$	0.89	0.04	0.11	0.19	$1.4 \cdot 10^{-3}$	0.61	0.40	$7.6 \cdot 10^{-3}$	0.35
**T1**	**AC**	**AE**	**CE**	**BD**	**BF**	**DF**	**AB**	**AD**	**AF**	**CB**	**CD**	**CF**	**EB**	**ED**	**EF**
h	0	0	0	0	0	0	0	0	0	0	0	0	0	0	0

p	0.05	0.03	0.72	0.04	0.97	0.08	0.96	0.06	0.74	0.03	0.98	0.07	0.01	0.59	0.03
**T2**	**AC**	**AE**	**CE**	**BD**	**BF**	**DF**	**AB**	**AD**	**AF**	**CB**	**CD**	**CF**	**EB**	**ED**	**EF**
h	**1**	**1**	**1**	0	0	1	0	1	0	1	1	1	0	0	1
p	**4.90 $\cdot 10^{-5}$**	**3.68 $\cdot 10^{-4}$**	**6.97 $\cdot 10^{-14}$**	0.42	2.7 $\cdot 10^{-3}$	8.34 $\cdot 10^{-4}$	0.04	3.73 $\cdot 10^{-4}$	0.82	9.33 $\cdot 10^{-13}$	2.44 $\cdot 10^{-13}$	1.05 $\cdot 10^{-5}$	0.49	0.92	2.24 $\cdot 10^{-4}$
**T3**	**AC**	**AE**	**CE**	**BD**	**BF**	**DF**	**AB**	**AD**	**AF**	**CB**	**CD**	**CF**	**EB**	**ED**	**EF**
h	0	0	0	**1**	**1**	0	1	1	1	0	1	1	1	1	1
p	1.2 $\cdot 10^{-3}$	0.60	0.01	**2.06 $\cdot 10^{-5}$**	**7.43 $\cdot 10^{-8}$**	0.26	1.12 $\cdot 10^{-9}$	4.57 $\cdot 10^{-23}$	5.25 $\cdot 10^{-28}$	0.01	1.53 $\cdot 10^{-11}$	2.97 $\cdot 10^{-15}$	2.38 $\cdot 10^{-7}$	6.98 $\cdot 10^{-19}$	2.31 $\cdot 10^{-23}$
Skeletonema marinoi **Kolmogorov-Smirnov**															
**T0**	**AC**	**AE**	**CE**	**BD**	**BF**	**DF**	**AB**	**AD**	**AF**	**CB**	**CD**	**CF**	**EB**	**ED**	**EF**
h	0	0	0	0	0	0	0	0	0	0	0	0	0	0	0
p	0.72	1	0.86	0.80	0.39	0.21	1	0.81	0.36	0.69	0.98	0.06	1	0.97	0.30
**T1**	**AC**	**AE**	**CE**	**BD**	**BF**	**DF**	**AB**	**AD**	**AF**	**CB**	**CD**	**CF**	**EB**	**ED**	**EF**
h	0	0	0	**1**	**1**	0	0	0	1	0	0	1	0	0	1
p	0.86	0.59	0.32	**8.79 $\cdot 10^{-4}$**	**1.08 $\cdot 10^{-4}$**	0.01	1	0.01	9.49 $\cdot 10^{-4}$	0.96	8.6 $\cdot 10^{-3}$	4.19 $\cdot 10^{-4}$	0.19	0.19	5.52 $\cdot 10^{-4}$
**T2**	**AC**	**AE**	**CE**	**BD**	**BF**	**DF**	**AB**	**AD**	**AF**	**CB**	**CD**	**CF**	**EB**	**ED**	**EF**
h	0	0	**1**	**1**	**1**	**1**	0	1	1	0	1	0	0	1	1
p	0.01	0.01	**2.61 $\cdot 10^{-6}$**	**1.48 $\cdot 10^{-19}$**	**3.47 $\cdot 10^{-3}$**	**2.52 $\cdot 10^{-10}$**	1	5.52 $\cdot 10^{-19}$	5.49 $\cdot 10^{-7}$	6.10 $\cdot 10^{-3}$	9.36 $\cdot 10^{-11}$	3.70 $\cdot 10^{-3}$	0.02	5.49 $\cdot 10^{-28}$	1.53 $\cdot 10^{-16}$
**T3**	**AC**	**AE**	**CE**	**BD**	**BF**	**DF**	**AB**	**AD**	**AF**	**CB**	**CD**	**CF**	**EB**	**ED**	**EF**
h	0	**1**	**1**	**1**	**1**	0	1	1	1	1	1	0	1	1	1
p	0.02	**1.46 $\cdot 10^{-6}$**	**1.87 $\cdot 10^{-12}$**	**1.61 $\cdot 10^{-29}$**	**2.18 $\cdot 10^{-23}$**	0.02	1.53 $\cdot 10^{-9}$	2.75 $\cdot 10^{-9}$	4.82 $\cdot 10^{-6}$	2.18 $\cdot 10^{-15}$	1.11 $\cdot 10^{-4}$	0.08	2.67 $\cdot 10^{-11}$	4.74 $\cdot 10^{-31}$	1.98 $\cdot 10^{-20}$
Skeletonema marinoi **Wilcoxon**															
**T0**	**AC**	**AE**	**CE**	**BD**	**BF**	**DF**	**AB**	**AD**	**AF**	**CB**	**CD**	**CF**	**EB**	**ED**	**EF**
h	0	0	0	0	0	0	0	0	0	0	0	0	0	0	0

p	0.68	0.95	0.77	0.70	0.20	0.07	0.94	0.76	0.18	0.63	0.88	0.06	0.89	0.81	0.15
**T1**	**AC**	**AE**	**CE**	**BD**	**BF**	**DF**	**AB**	**AD**	**AF**	**CB**	**CD**	**CF**	**EB**	**ED**	**EF**
h	0	0	0	0	0	0	0	0	0	0	0	1	0	0	0
p	0.60	0.98	0.57	0.51	$1.10 \cdot 10^{-3}$	$1.50 \cdot 10^{-3}$	0.55	0.96	$6.90 \cdot 10^{-3}$	0.93	0.46	$7.22 \cdot 10^{-4}$	0.53	0.86	$3.00 \cdot 10^{-3}$
**T2**	**AC**	**AE**	**CE**	**BD**	**BF**	**DF**	**AB**	**AD**	**AF**	**CB**	**CD**	**CF**	**EB**	**ED**	**EF**
h	0	0	**1**	**1**	**1**	**1**	0	1	1	0	1	0	0	1	1
p	0.01	$2.70 \cdot 10^{-3}$	**$1.01 \cdot 10^{-7}$**	**$4.79 \cdot 10^{-27}$**	**$9.11 \cdot 10^{-7}$**	**$3.01 \cdot 10^{-13}$**	0.83	$9.19 \cdot 10^{-26}$	$3.25 \cdot 10^{-6}$	$6.20 \cdot 10^{-3}$	$4.50 \cdot 10^{-15}$	0.09	$5.20 \cdot 10^{-3}$	$2.72 \cdot 10^{-38}$	$5.00 \cdot 10^{-15}$
**T3**	**AC**	**AE**	**CE**	**BD**	**BF**	**DF**	**AB**	**AD**	**AF**	**CB**	**CD**	**CF**	**EB**	**ED**	**EF**
h	0	**1**	**1**	**1**	**1**	0	1	1	1	1	1	0	0	1	1
p	0.08	**$1.73 \cdot 10^{-9}$**	**$1.01 \cdot 10^{-17}$**	**$3.70 \cdot 10^{-32}$**	**$4.07 \cdot 10^{-21}$**	$6.30 \cdot 10^{-3}$	$2.50 \cdot 10^{-8}$	$5.13 \cdot 10^{-12}$	$1.24 \cdot 10^{-5}$	$2.24 \cdot 10^{-13}$	$2.33 \cdot 10^{-8}$	$6.20 \cdot 10^{-3}$	0.32	$5.31 \cdot 10^{-45}$	$2.86 \cdot 10^{-29}$

Supplementary Table 9. **Non-parametric statistic tests** (Kolmogorov-Smirnov (KS2) and Wilcoxon) run in pairwise among turbulent vs still samples merging results from the three replicates. 'h' indicate the test answer: 0 = null hypothesis accepted; 1 = null hypothesis consistently rejected. 'p' indicate the p-value associated to the test outcome. Accuracy set at 99.9% ($\alpha = 10^{-3}$).

***Chaetoceros decipiens* Experiment 1**				
**KS2**	**T0**	**T1**	**T2**	
**h**	0	1	1	
**p**	1	$1.33 \cdot 10^{-4}$	$7.81 \cdot 10^{-15}$	
**Wilcoxon**	**T0**	**T1**	**T2**	
**h**	0	0	1	
**p**	0.58	$3.4 \cdot 10^{-3}$	$1.69 \cdot 10^{-16}$	
***Chaetoceros decipiens* Experiment 2**				
**KS2**	**T0**	**T1**	**T2**	**T3**
**h**	0	0	1	1
**p**	0.18	0.03	$1.56 \cdot 10^{-24}$	$4.24 \cdot 10^{-63}$
**Wilcoxon**	**T0**	**T1**	**T2**	**T3**
**h**	0	1	1	1
**p**	0.35	$5.78 \cdot 10^{-5}$	$5.78 \cdot 10^{-24}$	$4.35 \cdot 10^{-83}$
Thalassiosira rotula				
**KS2**	**T0**	**T1**	**T2**	**T3**
**h**	0	0	0	**1**
**p**	0.54	$1.0 \cdot 10^{-3}$	$3.60 \cdot 10^{-3}$	$2.69 \cdot 10^{-35}$
**Wilcoxon**	**T0**	**T1**	**T2**	**T3**
**h**	0	1	1	**1**
**p**	0.16	$4.88 \cdot 10^{-5}$	$6.28 \cdot 10^{-5}$	$3.15 \cdot 10^{-40}$

Supplementary Table 10. Incidence of broken chains in *Chaetoceros decipiens* experiments 1 and 2. The number of broken chains (BC) recorded during cell counts is reported for each TURBOGEN cylinder (turbulent condition A, C, E and still condition B, D, F). These numbers have been weighted over the total number of chains recorded in each sample (Percentage of BC). The overall percentage in turbulent and still conditions (red digits) was calculated as the sum of the BC recorded in each cylinder weighted over the total number of chains. These data were plotted in Fig. 4c (Experiment 1) and Fig. 4d (Experiment 2).

**Experiment 1**						
**Number of BC**						
**Time point**	**Turb**			**Still**		
	**A**	**C**	**E**	**B**	**D**	**F**
**T0**	5	1	1	1	2	1
**T1**	11	3	1	11	8	1
**T2**	11	4	6	15	22	14
**Percentage of BC**						
**%**	**Turb**			**Still**		
	**A**	**C**	**E**	**B**	**D**	**F**
**T0**	1.4	0.2	0.2	0.3	0.5	0.2
**T1**	3.4	0.8	0.2	2.9	2.2	0.2
**T2**	2.5	1.1	1.5	3.6	5.3	3.2
**Overall percentage of BC**						
**%**	**Turb**			**Still**		
**T0**	0.6			0.3		
**T1**	1.4			1.8		
**T2**	1.7			4.0		
**Experiment 2**						
**Number of BC**						
	**Turb**			**Still**		
	**A**	**C**	**E**	**B**	**D**	**F**
**T0**	2	1	3	1	3	2
**T1**	3	7	4	3	3	6
**T2**	15	17	13	20	19	20
**T3**	17	20	8	103	50	1
**Percentage of BC**						
**%**	**Turb**			**Still**		
	**A**	**C**	**E**	**B**	**D**	**F**
**T0**	0.5	0.2	0.7	0.2	0.7	0.5
**T1**	0.7	1.7	1.0	0.7	0.9	1.5
**T2**	3.7	4.2	3.5	4.7	4.4	5.0
**T3**	3.9	5.6	2.0	24.0	13.8	0.2
**Overall percentage of BC**						
**%**	**Turb**			**Still**		

**T0**	0.5	0.5
**T1**	1.2	1.0
**T2**	3.8	4.7
**T3**	3.8	12.8

Legend to the Supplementary Table 11
Supplementary Table 11 contains the first 50 hits from a tblastn search in the MMETSP database performed with transcripts listed in Supplementary Table 6.

Legend to the Supplementary Table 12
This file contains the annotation for the *Chaetoceros decipiens* transcriptome obtained from Annocript.

File **Supplementary Data 2** contains the nucleotide sequences of the *Chaetoceros decipiens* transcripts.

References

1 Ribera d'Alcalà, M. *et al.* Seasonal patterns in plankton communities in a pluriannual time series at a coastal Mediterranean site (Gulf of Naples): an attempt to discern recurrences and trends. *Sci. Mar.* 68, 65-83, doi:10.3989/scimar.2004.68s165 (2004).

2 Guillard, R. R. L. in *Culture of Marine Invertebrate Animals* (eds W.L. Smith & M.H. Chanley) 29-60 (Plenum Press, 1975).

3 Amato, A. *et al.* TURBOGEN: Computer-controlled vertically oscillating grid system for small-scale turbulence studies on plankton. *Rev. Sci. Instrum.* 87, 035119, doi:doi:http://dx.doi.org/10.1063/1.4944813 (2016).

4 Edler, L. & Elbrächter, M. in *Microscopic and molecular methods for quantitative phytoplankton analysis* (eds B. Karlson, C.K. Cusack, & E. Bresnan) 13-20 (UNESCO (IOC Manuals and Guides n. 55), 2010).

5 Bolger, A. M., Lohse, M. & Usadel, B. Trimmomatic: a flexible trimmer for Illumina sequence data. *Bioinformatics* 30, 2114-2120, doi:doi:10.1093/bioinformatics/btu170 (2014).

6 Haas, B. J. *et al.* *De novo* transcript sequence reconstruction from RNA-seq using the Trinity platform for reference generation and analysis. *Nat. Prot.* 8, 1494-1512, doi:10.1038/nprot.2013.084 (2013).

7 Langmead, B., Trapnell, C., Pop, M. & Salzberg, S. L. Ultrafast and memory-efficient alignment of short DNA sequences to the human genome. *Gen. Biol.* 10, R25, doi:10.1186/gb-2009-10-3-r25 (2009).

8 Musacchia, F., Basu, S., Petrosino, G., Salvemini, M. & Sanges, R. Annocript: a flexible pipeline for the annotation of transcriptomes able to identify putative long noncoding RNAs. *Bioinformatics* 31, 2199-2201, doi:10.1093/bioinformatics/btv106 (2015).

9 Altschul, S. F., Gish, W., Miller, W., Myers, E. W. & Lipman, D. J. Basic Local Alignment Search Tool. *J. Mol. Biol.* 215, 403-410, doi:10.1016/S0022-2836(05)80360-2. (1990).

10 Bairoch, A. & Apweiler, R. The SWISS-PROT Protein Sequence Data Bank and Its New Supplement TREMBL. *Nuc. Acids Res.* 24, 21-25, doi:10.1093/nar/24.1.21 (1996).

11 Suzek, B. E. *et al.* UniRef clusters: a comprehensive and scalable alternative for improving sequence similarity searches. *Bioinformatics* 31, 926-932, doi:10.1093/bioinformatics/btu739 (2015).

12 Marchler-Bauer, A. *et al.* CDD: NCBI’s Conserved Domain Database. *Nuc. Acids Res* 43, D222-D226, doi:10.1093/nar/gku1221 (2015).

13 Burge, S. W. *et al.* Rfam 11.0: 10 Years of RNA Families. *Nuc. Acids Res* 41, D226-D232, doi:doi:10.1093/nar/gks1005 (2012).

14 The Gene Ontology Consortium. The Gene Ontology project in 2008. *Nuc. Acids Res* 36, D440-D444, doi:10.1093/nar/gkm883 (2008).

15 Bairoch, A. The ENZYME Database in 2000. *Nuc. Acids Res* 28, 304-305, doi:10.1093/nar/28.1.304 (2000).

16 Morgat, A. *et al.* UniPathway: A Resource for the Exploration and Annotation of Metabolic Pathways. *Nuc. Acids Res* 40, D761-D769, doi:10.1093/nar/gkr1023 (2012).

17 Robinson, M. D., McCarthy, D. J. & Smyth, G. K. edgeR: A Bioconductor Package for Differential Expression Analysis of Digital Gene Expression Data. *Bioinformatics* 26, 139-140, doi:10.1093/bioinformatics/btp616 (2010).

18 Dolch, L.-J. & Maréchal, E. Inventory of fatty acid desaturases in the pennate diatom *Phaeodactylum tricornutum*. *Mar. Drugs* **13**, 1317-1339, doi:10.3390/md13031317 (2015).

19 Bernardes, J., Zaverucha, G., Vaquero, C. & Carbone, A. Improvement in protein domain identification is reached by breaking consensus, with the agreement of many profiles and domain co-occurrence. *PLoS Comput. Biol.* **12**, e1005038, doi:10.1371/journal.pcbi.1005038 (2016).
